# Briavioids E–G, Newly Isolated Briarane-Diterpenoids from a Cultured Octocoral *Briareum violaceum*

**DOI:** 10.3390/md21020124

**Published:** 2023-02-14

**Authors:** Thanh Hao Huynh, Chia-Jung Liu, Yi-Hung Liu, Su-Ying Chien, Zhi-Hong Wen, Lee-Shing Fang, Jih-Jung Chen, Yang-Chang Wu, Jui-Hsin Su, Ping-Jyun Sung

**Affiliations:** 1 Department of Marine Biotechnology and Resources, National Sun Yat-sen University, Kaohsiung 804201, Taiwan; 2 National Museum of Marine Biology and Aquarium, Pingtung 944401, Taiwan; 3 Instrumentation Center, National Taiwan University, Taipei 106319, Taiwan; 4 Institute of BioPharmaceutical Sciences, National Sun Yat-sen University, Kaohsiung 804201, Taiwan; 5 Center for Environmental Toxin and Emerging-Contaminant Research, Cheng Shiu University, Kaohsiung 833301, Taiwan; 6 Super Micro Mass Research and Technology Center, Cheng Shiu University, Kaohsiung 833301, Taiwan; 7 Faculty of Pharmacy, School of Pharmaceutical Sciences, National Yang Ming Chiao Tung University, Taipei 112304, Taiwan; 8 Graduate Institute of Integrated Medicine, College of Chinese Medicine, China Medical University, Taichung 404333, Taiwan; 9 Chinese Medicine Research and Development Center, China Medical University Hospital, Taichung 404394, Taiwan; 10 Graduate Institute of Natural Products, Kaohsiung Medical University, Kaohsiung 807378, Taiwan; 11 Ph.D. Program in Pharmaceutical Biotechnology, Fu Jen Catholic University, New Taipei City 242062, Taiwan

**Keywords:** *Briareum violaceum*, briarane, briavioid, X-ray, anti-inflammation, iNOS, COX-2

## Abstract

The chemical screening of a cultured soft coral, *Briareum violaceum*, led to the isolation of eight natural, briarane-related diterpenoids, including three unreported metabolites, briavioids E–G (**1**–**3**), and five known briaranes, briacavatolides B (**4**) and C (**5**), briaexcavatin L (**6**), briaexcavatolide U (**7**) and briarenol K (**8**). The structures of briaranes **1**–**8** were established using spectroscopic methods. The absolute configuration of briavioid A (**9**), obtained in a previous study, was reported for the first time in this study by a single-crystal X-ray diffraction analysis using a copper radiation source. The anti-inflammatory activity of briaranes **1** and **2** and briaranes **4**–**8** was evaluated by screening their inhibitory ability against the expression of inducible nitric oxide synthase (iNOS) and cyclooxygenase-2 (COX-2) proteins in lipopolysaccharide (LPS)-induced RAW 264.7 macrophage cells.

## 1. Introduction

**Figure 1:**
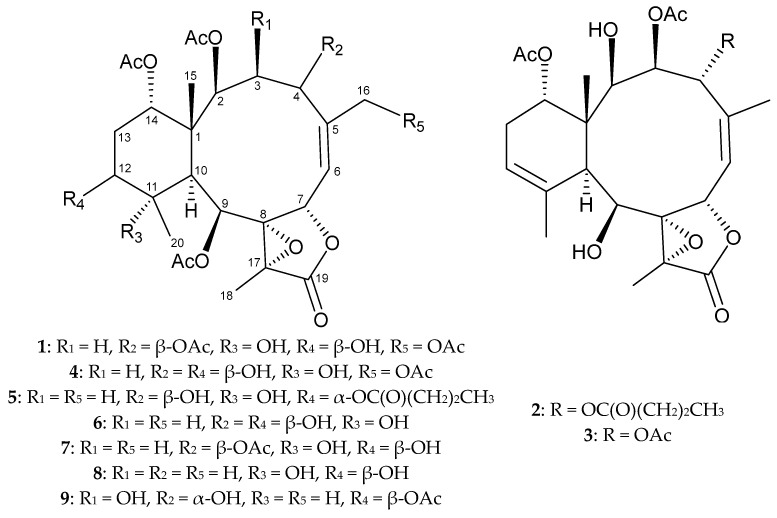
Structures of briavioids E–G (**1**–**3**), briacavatolides B (**4**) and C (**5**), briaexcavatin L (**6**), briaexcavatolide U (**7**), briarenol K (**8**) and briavioid A (**9**).

Every year, thousands of newly isolated marine natural products are reported [1,2]. These metabolites provide a wide range of bioactivities, including anti-cancer, anti-viral, anti-bacterial, anti-fungal and anti-inflammatory abilities [1,2]. These natural products are primarily isolated from marine microorganisms, algae and invertebrates. In this study, we describe the continuing work on the exploration of new substances from cultured marine invertebrates, which may possess interesting bioactivities. Here, eight natural briaranes were obtained from a cultured octocoral, *Briareum violaceum* (Quoy & Gaimard, 1833) (phylum: Cnidaria, sub-phylum: Anthozoa, class: Octocorallia, order: Scleralcyonacea, family: Briareidae) [3,4,5], including three unreported metabolites, briavioids E–G (**1**–**3**), and five reported analogues, briacavatolides B (**4**) and C (**5**) [6], briaexcavatin L (**6**) [7], briaexcavatolide U (**7**) [8] and briarenol K (**8**) [9] (Figure 1). Briaranes are a type of 3,8-cyclized cembranoid found only in marine invertebrates [10]. Most compounds of this type contain a bicyclo [8.4.0] system and a γ-lactone moiety in their structures and have a potential anti-inflammatory activity [11]. We reported herein the isolation and structural determination of all isolates, as well as the anti-inflammatory profile of compounds **1**, **2** and **4**–**8** using an in vitro assay to screen the reducing ability of these compounds against iNOS and COX-2 protein expression. In addition, the absolute configuration of briavioid A (**9**) [12] (Figure 1), obtained in previous study, was further reported for its absolute stereochemistry using a single-crystal X-ray diffraction analysis with a diffractometer equipped with a copper radiation (Cu Kα) source.

## 2. Results and Discussion

Briavioid E (**1**) was obtained as an amorphous powder with a molecular formula determined to be C_30_H_40_O_15_ by positive-mode, high-resolution electrospray ionization mass spectrum [(+)-HRESIMS] at *m*/*z* 663.22570 (calculated for C_30_H_40_O_15_ + Na, 663.22594), corresponding to 11 double-bond equivalents (DBEs). The IR spectrum of **1** showed absorptions at *ν*_max_ 3465, 1782 and 1735 cm^–1^, consistent with the presence of hydroxy, γ-lactone and ester moieties, respectively. The ^1^H and ^13^C NMR data of **1** (Table 1), in combination with the DEPT and HSQC spectra, revealed the presence of five acetoxy groups (*δ*_H_ 2.01, 2.03, 2.05, 2.10, 2.25/*δ*_C_ 21.3, 21.1, 20.8, 21.0, 21.3, 5 × acetate methyl; *δ*_C_ 170.5, 170.4, 169.8, 170.0, 167.9 and 5 × acetate carbonyl), two exchangeable protons (*δ*_H_ 1.58, 1H, s, OH-11; 2.14, 1H, d, *J* = 2.8 Hz, OH-12), a trisubstituted carbon–carbon double bond (*δ*_C_ 140.9, C-5; *δ*_H_ 5.48, 1H, ddd, *J* = 8.4, 1.6, 1.6 Hz/*δ*_C_ 122.9, CH-6), a diastereotopic oxymethylene (*δ*_H_ 4.76, 1H, dd, *J* = 15.6, 1.6 Hz; 5.25, 1H, dd, *J* = 15.6, 1.6 Hz/*δ*_C_ 65.7, CH_2_-16), a γ-lactone carbonyl (*δ*_C_ 170.1, C-19), and three *sp*^3^ tertiary oxygenated carbons, an *sp*^3^ quaternary non-oxygenated carbon, six *sp*^3^ oxymethines, an *sp*^3^ aliphatic methine, two *sp*^3^ aliphatic methylenes and three methyls (Table 1). Considering the functional groups observed for **1**, the presence of a tetracyclic ring system in **1** was inferred.

**Figure 2:**
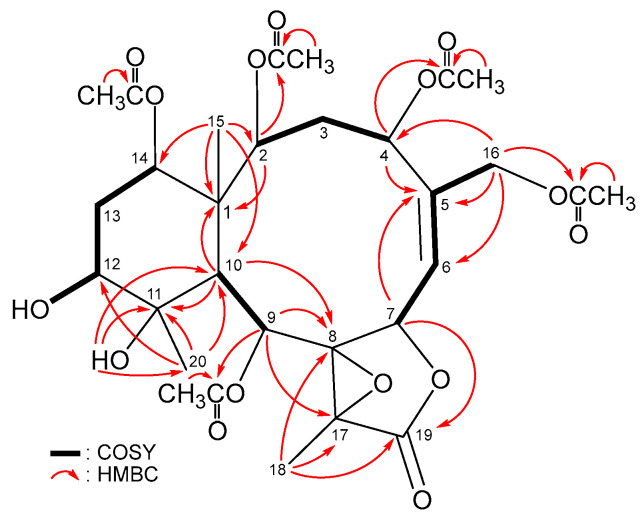
Key COSY and HMBC correlations of **1**.

The ^3^*J*-proton–proton coupling information from the ^1^H–^1^H COSY spectrum revealed four continuous spin systems from H-2/H_2_-3/H-4, H-6/H-7, H-9/H-10 and H-12/H_2_-13/H-14 (Figure 2) in **1**. The HMBC spectrum showed ^2^*J*- and ^3^*J*-heteronuclear correlations from neighbor protons to the non-protonated carbons, including H-2, H-10, H_3_-15/C-1; H-4, H-7, H_2_-16/C-5; H-9, H-10, H_3_-18/C-8; H-10, H_3_-20/C-11; H-9, H_3_-18/C-17 and H-7, H_3_-18/C-19 (Figure 2), thus establishing the tetracyclic 10/6/5/3 ring system of the briarane scaffold. The HMBC correlations from H_3_-15/C-1, C-2, C-10, C-14; H_3_-18/C-8, C-17, C-19 and H_3_-20/C-10, C-11, C-12 suggested that Me-15, Me-18 and Me-20 are at C-1, C-17 and C-11, respectively. An acetoxymethyl at C-5 was illustrated by the HMBC correlations from H_2_-16 to C-4, C-5, C-6 and by the long-range allylic couplings between H_2_-16 and H-6 (*J* = 1.6, 1.6 Hz) (Figure 2). HMBC correlations, observed from a hydroxy proton at *δ*_H_ 1.58 (OH-11) to C-10, C-11 and C-20, and the COSY correlation between a hydroxy proton at *δ*_H_ 2.14 (OH-12) and H-12 indicate that these two hydroxy groups are placed at C-11 and C-12, respectively. The H-2 (*δ*_H_ 4.97), H-4 (*δ*_H_ 4.99), H-9 (*δ*_H_ 5.80) and H_2_-16 (*δ*_H_ 4.76 and 5.25) correlations to the acetate carbonyls at *δ*_C_ 170.5, 169.8, 167.9 and 170.0 confirmed that these four acetoxy groups are positioned at C-2, C-4, C-9 and C-16, respectively. Based on the chemical shifts of oxymethine CH-14 (*δ*_C_ 74.6/*δ*_H_ 4.83, 1H, dd, *J* = 3.6, 2.0 Hz), the remaining acetoxy group is OAc-14. Fourteen of the fifteen oxygen atoms in the molecular formula of briarane **1** could be accounted for from the presence of one γ-lactone, five esters and two hydroxy groups. The remaining single oxygen atom had to be placed between C-8 and C-17 to form a tetrasubstituted epoxide containing a methyl substituent, based on the ^13^C NMR evidence of two tertiary oxygenated carbons at *δ*_C_ 70.4 (C-8) and 66.1 (C-17) and from the chemical shifts of a tertiary methyl (*δ*_H_ 1.79, 3H, s/*δ*_C_ 10.2, CH_3_-18). Thus, the planar structure of **1**, including the positions of all functionalities, was fully determined.

**Figure 3:**
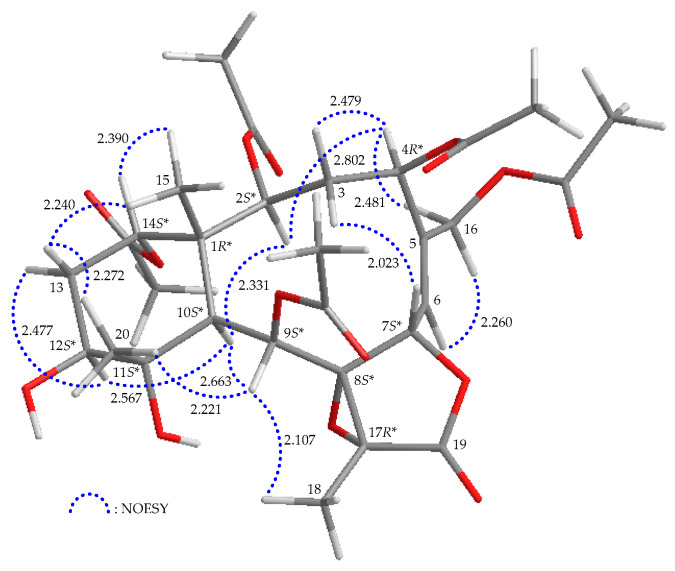
Stereoview of **1** and calculated distances (Å) between particular protons that have crucial NOESY correlations.

The relative stereochemistry of briarane **1** was established by interpreting the NOESY spectrum in addition to the assistance of the computer-generated modeling structure. The literature review indicated that most naturally isolated briaranes have a β-Me and an α-H placed at C-1 and C-10, respectively [10]. From the NOESY data of **1** (Figure 3), H_3_-15 exhibited cross-peaks with H-14 and one of the diastereotopic methylene protons at C-13 (*δ*_H_ 1.67, H-13β) but not with H-10; while H-10 was correlated to H-2, H-9 and H-12, consistent with the α-orientation of OAc-14 and β-orientations of OAc-2, OAc-9 and OH-12, respectively. Furthermore, H_3_-20 showed a correlation to H-13β and the absence of cross-peaks with H-10 and H-12, suggesting a β-oriented methyl group at C-11. One of the C-3 diastereotopic methylene protons (*δ*_H_ 2.97) exhibited a correlation to H-7 but not to H-2 and H-4, suggesting the β-orientations of this proton and H-7. The other was assigned as H-3α (δ_H_ 2.04). H-4 showed a correlation with H-3α, and a greater coupling constant of 12.4 Hz was noted between H-4 and H-3β, demonstrating the α-orientation of H-4 [13,14] and that the plane between H-4 and H-3β has a dihedral angle of approximately 180°. H-9 correlated to H-10, H_3_-18 and H_3_-20, suggesting that H-9 is close to all these protons. In combination with model analysis, Me-18 and 8,17-epoxy group should be placed at β- and α-face in the γ-lactone moiety, respectively. A correlation between H-6 and a proton of the C-16 diastereotopic methylene (*δ*_H_ 5.25) but not with H-7, in addition to a large coupling constant between H-7 and H-6 (*J* = 8.4 Hz), suggested that the dihedral angle between H-7 and H-6 was nearly 130° [13,14], revealing the *Z*-geometry of ∆^5^. The above interpretation enables the identification the relative configuration of all stereogenic centers of **1** as (1R*,2S*,4R*,7S*,8S*,9S*,10S*,11S*,12S*,14S*,17R*).

**Figure 4:**
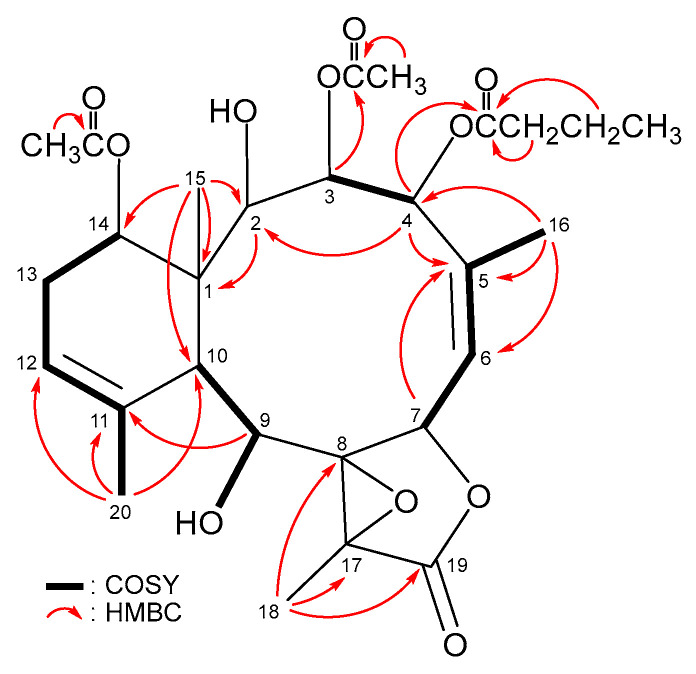
Key COSY and HMBC correlations of **2**.

Briavioid F (**2**) was isolated as an amorphous powder. Its HRESIMS peak was at *m*/*z* 573.23086, consistent with the molecular formula C_28_H_38_O_11_ (calculated for C_28_H_38_O_11_ + Na, 573.23063) with 10 degrees of unsaturation. The IR spectrum of **2** contained signals of hydroxy (*ν*_max_ 3293 cm^–1^), γ-lactone (*ν*_max_ 1785 cm^–1^) and ester (*ν*_max_ 1735 cm^–1^) functionalities. Analyzing the ^1^H, ^13^C, DEPT and HSQC spectra of **2** led to the assignment of two acetoxy (*δ*_H_ 2.03, 2.09, both 3H × s/*δ*_C_ 20.7, 22.0, two acetate methyls; *δ*_C_ 168.9, 170.7; and two acetate carbonyls), an *n*-butyroxy (*δ*_C_ 174.3, *n*-butyrate carbonyl; *δ*_H_ 2.37, 1H, dt, *J* = 16.4, 7.6 Hz and 2.28, 1H, dt, *J* = 16.4, 7.6 Hz/*δ*_C_ 35.8, CH_2_; *δ*_H_ 1.65, 2H, sext, *J* = 7.6 Hz/*δ*_C_ 18.1, CH_2_; *δ*_H_ 0.97, 3H, t, *J* = 7.6 Hz/*δ*_C_ 13.7, CH_3_) and two hydroxy (*δ*_H_ 5.38, 1H, br s, OH-2; 6.45, 1H, br d, *J* = 6.4 Hz, OH-9) groups; as well as two trisubstituted carbon–carbon double bonds (*δ*_C_ 137.7, C-5; *δ*_H_ 5.65, 1H, dd, *J* = 10.0, 1.2 Hz/*δ*_C_ 126.1, CH-6; *δ*_C_ 134.3, C-11; *δ*_H_ 5.34, 1H, dd, *J* = 5.2, 0.8 Hz/*δ*_C_ 119.6, CH-12), a γ-lactone moiety (*δ*_C_ 172.1, C-19), a tetrasubstituted epoxide (*δ*_C_ 71.1, C-8; 61.3, C-17) and other 13 carbon signals (Table 1). The carbon-skeleton of **2**, including the positions of the two trisubstituted olefins and the tetrasubstituted epoxide, was fully established by following correlations observed in the COSY and HMBC spectra (Figure 4). The oxymethine protons H-3 (*δ*_H_ 5.71) and H-4 (*δ*_H_ 6.32) showed HMBC correlations to the acetate carbonyl at *δ*_C_ 168.9 and *n*-butyrate carbonyl at *δ*_C_ 174.3, confirmed the position of acetoxy and *n*-butyroxy groups at C-3 and C-4, respectively. H-9 (*δ*_H_ 3.94) correlated to a hydroxy proton resonating at *δ*_H_ 6.45 in the COSY spectrum, suggesting a hydroxy group at C-9. Evaluated on the chemical shifts of H-2 (*δ*_H_ 3.88) and H-14 (*δ*_H_ 4.60), the remaining hydroxy and acetoxy groups should be positioned at C-2 and C-14, respectively.

**Figure 5:**
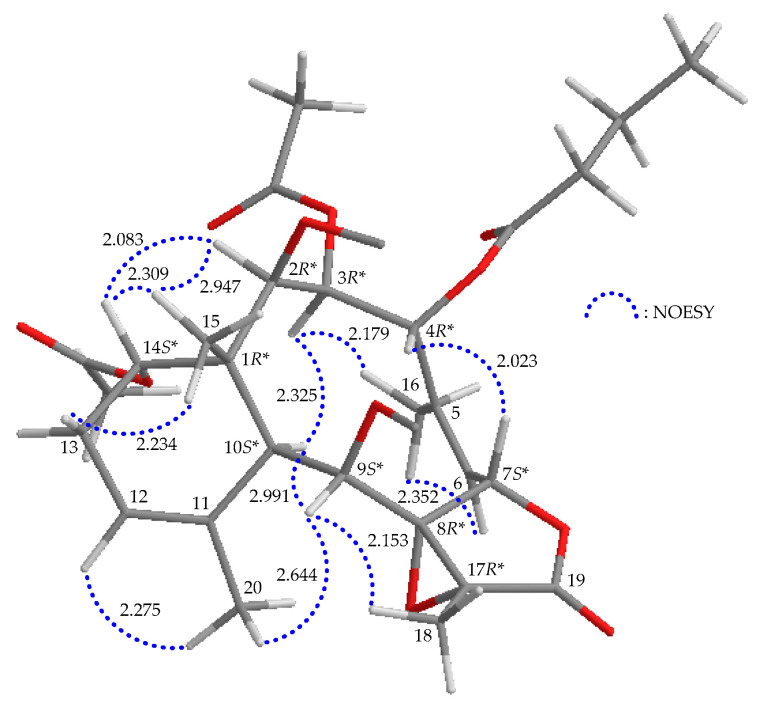
Stereoview of **2** and calculated distances (Å) between particular protons that have crucial NOESY correlations.

In the NOESY data of **2** (Figure 5), H_3_-15 correlated with H-14 and H-10 correlated with H-3 and H-9, illustrating the α-orientation of OAc-14 and β-orientations of Oac-3 and OH-9. H-2 lacks a coupling with H-3, consistent with the dihedral angle of these two protons being approximately 90° [13,14], and H-2 showed NOE effects with both H-14 and H_3_-15, indicating that the conformation of H-2 is α-oriented. H-4 correlated with H-7 but not with H-3 and H_3_-16; with the assistance of the modeling structure, both H-4 and H-7 should be oriented to β-face. The olefin protons H-6 and H-12 showed correlations with H_3_-16 and H_3_-20, respectively, confirming the *Z*-geometries of ∆^5^ and ∆^11^. H-9 correlated to H-10, H_3_-18 and H_3_-20 in the NOESY spectrum, suggesting that these protons are close in space, implying that, in the computer-generated model structure, the Me-18 and 8,17-epoxide should be placed at the β- and α-face of the γ-lactone moiety, respectively. Therefore, based on the above findings, the relative stereochemistry of briavioid F (**2**) was established as (1R*,2R*,3R*,4R*,7S*,8R*,9S*,10S*,14S*,17R*).

Briavioid G (**3**) was isolated as an amorphous powder. The molecular formula of **3** was determined as C_26_H_34_O_11_ (10 degrees of unsaturation) based on a positive ion peak at *m*/*z* 545.19912, which presented in its HRESIMS spectrum (calculated for C_26_H_34_O_11_ + Na, 545.19933). The IR signals of this compound suggested the presences of hydroxy (*ν*_max_ 3304 cm^–1^), γ-lactone (*ν*_max_ 1784 cm^–1^) and ester (*ν*_max_ 1741 cm^–1^) groups. The ^1^H and ^13^C NMR data of **3** were found to be almost identical to those of **2** (Table 1), except the signals of a *n*-butyroxy group in **2** were replaced by the signals for an acetoxy group in **3**, indicating that these two compounds different only on the functional group at C-4.

**Figure 6:**
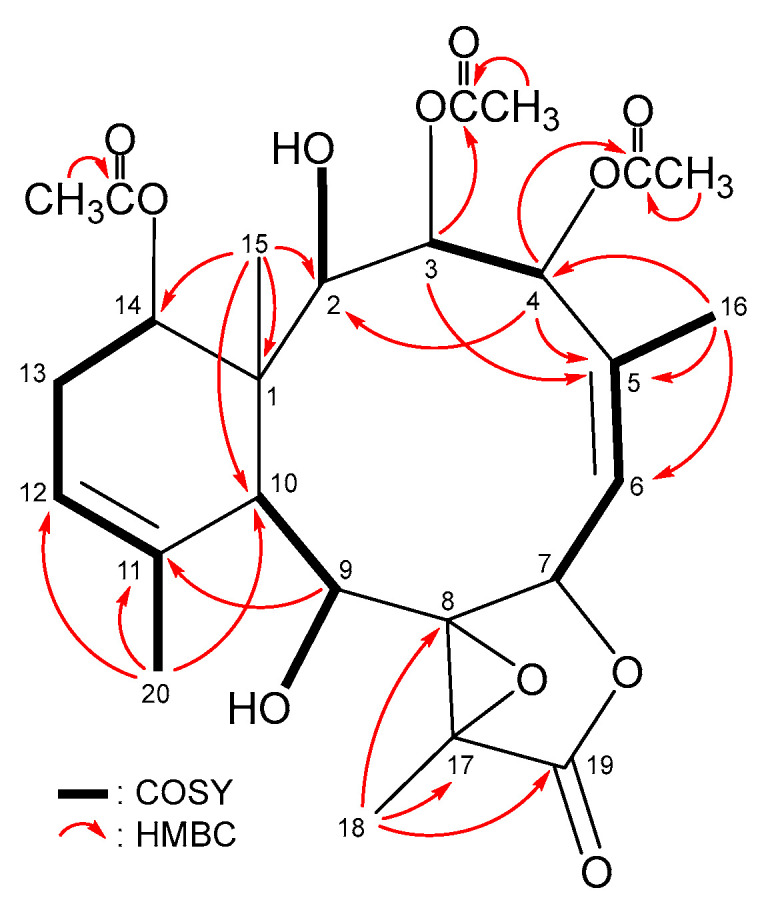
Key COSY and HMBC correlations of **3**.

**Figure 7:**
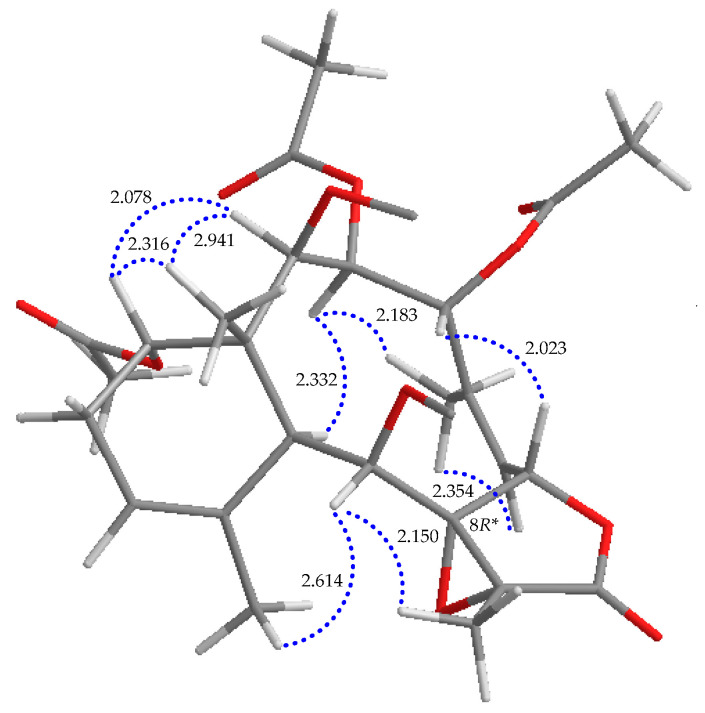
Stereoview of **3** and calculated distances (Å) between particular protons that have crucial NOESY correlations.

The planar structure of **3**, including the positions of OAc-3, OAc-4, OAc-14, OH-2 and OH-9 then was clearly confirmed by analyzing the COSY and HMBC spectroscopic data (Figure 6). Based on the NMR data of **2** and **3**, the stereochemistry of **3** should be similar to the configuration of **2**. It then was further confirmed by the combined interpretation of the NOESY correlation and modeling structure (Figure 7). The configuration of **3** was identified as (1R*,2R*,3R*,4R*,7S*,8R*,9S*,10S*,14S*,17R*).

**Figure 8:**
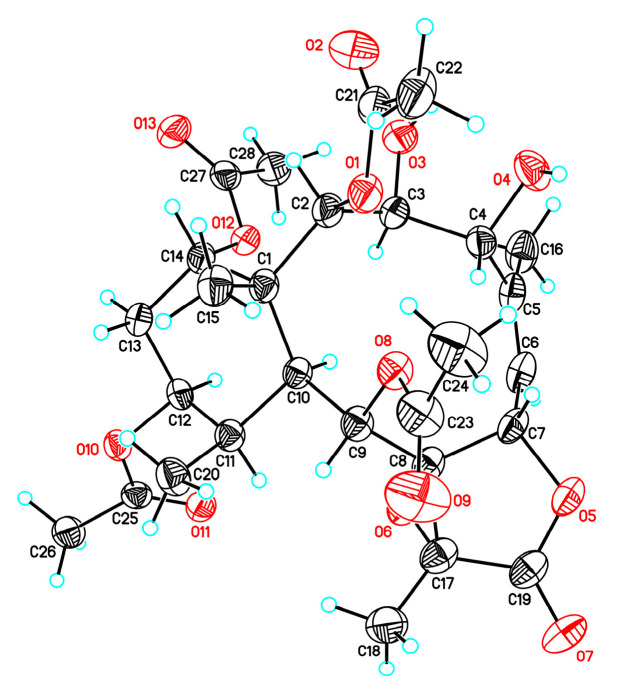
The ORTEP diagram of briavioid A (**9**).

Briavioid A (**9**) was reported in our previous publication [12]. Its relative stereochemistry was established through a combination of the NOESY experiment and a single-crystal X-ray diffraction analysis with a molybdenum radiation (Mo Kα, λ *=* 0.71073 Å) source. However, the configuration obtained from the X-ray analysis with Mo Kα could only be considered absolute when the structure contained at least one heavy atom. Thus, in order to determine the absolute configuration of this compound, the material of briavioid A (**9**) obtained in previous publication [12] was recrystallized, and the diffraction experiment was carried out using a diffractometer equipped with Cu Kα (λ *=* 1.54178 Å) radiation source (Flack parameter x = 0.01(5)). An Oak Ridge Thermal-Ellipsoid Plot (ORTEP) diagram (Figure 8) showed the absolute configurations of the stereogenic carbons of **9** to be (1*R*,2*R*,3*S*,4*R*,7*S*,8*S*,9*S*,10*S*,11*R*,12*S*,14*S*,17*R*).

As briaranes **1**–**3**, in addition to briavioid A (**9**), were isolated from the same target organism, *B. violaceum*, it is reasonable to assume on biogenetic grounds that briaranes **1**–**3** have the same absolute configurations as **9**. Therefore, the absolute configurations of **1**–**3** were suggested to be (1*R*,2*S*,4*R*,7*S*,8*S*,9*S*,10*S*,11*S*,12*S*,14*S*,17*R*), (1*R*,2*R*,3*R*,4*R*,7*S*,8*R*,9*S*, 10*S*,14*S*,17*R*) and (1*R*,2*R*,3*R*,4*R*,7*S*,8*R*,9*S*,10*S*,14*S*,17*R*), respectively.

The known briaranes **4**–**8** were identified as briacavatolides B and C, briaexcavatin L, briaexcavatolide U and briarenol K, respectively, based on their spectroscopic data. Including IR, ESIMS, ^1^H, ^13^C and DEPT NMR data, they were found to be identical with those of the reported data [6,7,8,9].

The anti-inflammation profiles of briaranes **1** and **2** and **4**–**8** were screened using the in vitro pro-inflammatory assay to test the inhibitory ability of these compounds against the iNOS and COX-2 protein expressions in LPS-induced RAW 264.7 macrophage cells. The results are shown in Table 2. Except for briarane **6** (briaexcavatin L), all isolates exhibited moderate activity to suppress the generation of iNOS but were not active in the inhibition of COX-2. Even briaranes **2** (briavioid F), **7** (briaexcavatolide U) and **8** (briarenol K) exhibited activity to enhance the release of COX-2. It is interesting to note that the most active compound, **2**, significantly reduced the release of iNOS to 51.60% at a concentration of 10 μM; however, this compound enhanced the generation of COX-2 to 140.73%. Briaranes **4** (briacavatolide B) and **7** were also found to display a moderate inhibition effect toward iNOS. Briarane **6** did not show activity toward iNOS, implying that the presence of an acetoxy group at C-4 or C-16 would enhance the activity in comparison with the structure and anti-inflammatory activity of **4** and **7**.

## 3. Materials and Methods

### 3.1. General Experimental Procedures

The specific rotation values and IR spectra were measured using a JASCO P-2000 digital polarimeter and a THERMO Scientific Nicolet iS5 FT-IR spectrophotometer, respectively. ESIMS and HRESIMS were recorded using a BRUKER 7 Tesla solariX FTMS system. NMR spectra were obtained from a JEOL Resonance ECZ 400S or an ECZ 600R NMR spectrometer, with the residual signals of CHCl_3_ (*δ*_H_ 7.26 ppm) and CDCl_3_ (*δ*_C_ 77.0 ppm) used as the internal standards for ^1^H and ^13^C NMR, respectively. Coupling constants (*J*) are provided in Hz. Column chromatography was carried out with a silica gel (230~400 mesh, MERCK) column. Thin-layer chromatography was performed on plates precoated with silica gel 60 F_254_ (0.25-mm-thick, MERCK); the plates then sprayed with 10% (*v*/*v*) H_2_SO_4_ in methanol, followed by heating to visualize the spots. A normal-phase (NP) HPLC was performed using a system comprised of a HITACHI 5110 pump, a RHEODYNE 7725i injection port and a NP column (YMC pack SIL, 5 μm, 12 nm, 250 × 20 mm, YMC group). Reverse-phase (RP) HPLC was performed using a system comprised of a HITACHI L-2130 pump, a HITACHI L-2455 photodiode array detector, a RHEODYNE 7725i injection port and a RP column (Luna 5 µm C18(2) 100 Å, 250 × 21.2 mm, Phenomenex).

### 3.2. Animal Material

The studied organism was cultured by the National Museum of Marine Biology & Aquarium (NMMBA), Taiwan, in an 80 ton culturing tank. Specimens were collected from the tank in December 2016, and the organism was identified through a comparison of the morphology and the micrograph of sclerites with published, scientific descriptions of *Briareum violaceum* [3,4,5]. A voucher specimen was deposited in the NMMBA (NMMBA-CSC-002).

### 3.3. Extraction and Isolation

The detailed extraction procedures for the crude extract and the ethyl acetate extract used in this study are described in our previous publication [12]. The EtOAc extract (31.2 g) was applied to a silica gel open column and eluted with gradients of hexanes/EtOAc (100% hexanes~100% ethyl acetate, stepwise), resulting in 11 fractions (fractions A–K). Fraction G (120 out of 1800 mg) was injected into a NP-HPLC column and run with a mixture of *n*-hexane/acetone (2:1, flow rate = 5 mL/min) to afford subfractions G1–G7. Subfractions G3 and G4 then were purified by RP-HPLC (60% MeOH in H_2_O, flow rate = 5 mL/min for G3; 65% MeOH in H_2_O, flow rate = 4 mL/min for G4) to obtain **8** (11.0 mg) and **5** (4.5 mg) from G3, and **7** (4.0 mg) from G4, respectively. Fraction H (90 out of 700 mg) was injected into a NP-HPLC column and run with a mixture of *n*-hexane/acetone (2/1, flow rate = 5 mL/min) to afford subfractions H1–H7, including **6** (20.0 mg). Subfractions H1 and H4 then were further separated by RP-HPLC (60% MeOH in H_2_O, flow rate = 4 mL/min) to obtain **4** (1.2 mg) and **1** (0.7 mg), respectively. Fraction I (100 out of 300 mg) was injected into a NP-HPLC column and run with a mixture of *n*-hexane/acetone (4.5/2, flow rate = 5 mL/min) to afford subfractions I1–I6. Subfraction I2 was then purified by RP-HPLC (80% MeOH in H_2_O, flow rate = 4 mL/min) to obtain **2** (0.6 mg) and **3** (0.2 mg), respectively.

Briavioid E (**1**): Amorphous powder; [α]${}_{D}^{24}$–140 (*c* 0.04, CHCl_3_); IR (ATR) *ν*_max_ 3465, 1782, 1735 cm^–1^; ^1^H (400 MHz, CDCl_3_) and ^13^C (100 MHz, CDCl_3_) NMR data, see Table 1; ESIMS *m*/*z* 663 [M + Na]^+^; HRESIMS *m*/*z* 663.22570 (calculated for C_30_H_40_O_15_ + Na, 663.22594).

Briavioid F (**2**): Amorphous powder; [α]${}_{D}^{24}$–27 (*c* 0.03, CHCl_3_); IR (ATR) *ν*_max_ 3293, 1785, 1735 cm^–1^; ^1^H (400 MHz, CDCl_3_) and ^13^C (100 MHz, CDCl_3_) NMR data, see Table 1; ESIMS *m*/*z* 573 [M + Na]^+^; HRESIMS *m*/*z* 573.23086 (calculated for C_28_H_38_O_11_ + Na, 573.23063).

Briavioid G (**3**): Amorphous powder; [α]${}_{D}^{24}$–36 (*c* 0.01, CHCl_3_); IR (ATR) *ν*_max_ 3304, 1784, 1741 cm^–1^; ^1^H (600 MHz, CDCl_3_) and ^13^C (150 MHz, CDCl_3_) NMR data, see Table 1; ESIMS *m*/*z* 545 [M + Na]^+^; HRESIMS *m*/*z* 545.19912 (calculated for C_26_H_34_O_11_ + Na, 545.19933).

Briacavatolide B (**4**): Amorphous powder; [α]${}_{D}^{24}$–33 (*c* 0.06, CHCl_3_) (ref. [6] [α]${}_{D}^{25}$ –57.8 (*c* 0.1, CHCl_3_)); IR (ATR) *ν*_max_ 3447, 1780, 1734 cm^–1^; ^1^H and ^13^C NMR data were found to be in agreement with a previous publication [6]; ESIMS *m*/*z* 621 [M + Na]^+^.

Briacavatolide C (**5**): Amorphous powder; [α]${}_{D}^{24}$+72 (*c* 0.22, CHCl_3_) (ref. [6] [α]${}_{D}^{25}$ +25.5 (*c* 0.1, CHCl_3_)); IR (ATR) *ν*_max_ 3492, 1781, 1735 cm^–1^; ^1^H and ^13^C NMR data were found to be in agreement with a previous publication [6]; ESIMS *m*/*z* 633 [M + Na]^+^.

Briaexcavatin L (**6**): Amorphous powder; [α]${}_{D}^{24}$+10 (*c* 0.9, CHCl_3_) (ref. [7] [α]${}_{D}^{25}$ +72 (*c* 0.22, CHCl_3_)); IR (ATR) *ν*_max_ 3447, 1772, 1734 cm^–1^; ^1^H and ^13^C NMR data were found to be in agreement with a previous publication [7]; ESIMS *m*/*z* 563 [M + Na]^+^.

Briaexcavatolide U (**7**): Amorphous powder; [α]${}_{D}^{24}$+213 (*c* 0.2, CHCl_3_) (ref. [8] [α]${}_{D}^{25}$ +48 (*c* 0.1, CHCl_3_)); IR (ATR) *ν*_max_ 3482, 1781, 1735 cm^–1^; ^1^H and ^13^C NMR data were found to be in agreement with a previous publication [8]; ESIMS *m*/*z* 605 [M + Na]^+^.

Briarenol K (**8**): Amorphous powder; [α]${}_{D}^{24}$+83 (*c* 0.2, CHCl_3_) (ref. [9] [α]${}_{D}^{25}$ +37 (*c* 0.06, CHCl_3_)); IR (ATR) *ν*_max_ 3476, 1774, 1735 cm^–1^; ^1^H and ^13^C NMR data were found to be in agreement with a previous publication [9]; ESIMS *m*/*z* 547 [M + Na]^+^.

### 3.4. Single-Crystal X-ray Crystallography of Briavioid A *(**9**)*

Suitable, colorless prisms of briavioid A (**9**) were obtained from a mixture of MeOH/acetone (10:1). The crystal (0.400 × 0.050 × 0.025 mm^3^) was identified as of the monoclinic system, space group *P*2_1_ (#4), with *a* = 9.8798(6) Å, *b* = 31.107(2) Å, *c* = 10.4985(7) Å, *V* = 2871.5(3) Å^3^, *Z* = 4, *D*_calculated_ = 1.348 Mg/m^3^ and *λ* (Cu K*α*) = 1.54178 Å. Intensity data were obtained on a crystal diffractometer (Bruker, AXS D8 Venture, Photon III_C28) up to a *θ*_max_ of 77.86°. All measurement data from 60,292 reflections were collected, of which 10,314 were independent. The structure was solved by direct methods and refined by a full-matrix least-squares on *F*^2^ procedure [15,16]. The refined structural model converged to a final R_1_ = 0.0427; wR_2_ = 0.1145 for 9539 observed reflections [*I* > 2σ(*I*)] and 759 variable parameters. The absolute configuration was established from the Flack parameter x = 0.01(5) [17,18]. Crystallographic data for the structure of briavioid D (**9**) were deposited at the Cambridge Crystallographic Data Center (CCDC) as supplementary publication number CCDC 2224010 [19].

### 3.5. In Vitro Anti-Inflammatory Assay

The anti-inflammatory activity of briaranes **1** and **2** and **4**–**8** was tested by evaluating their inhibitory ability against the expression of iNOS and COX-2 pro-inflammatory proteins in LPS-induced RAW 264.7 macrophage cells. The method was described in detail in the previous publications [20,21].

## 4. Conclusions

In this study, the chemical composition of a cultured octocoral, identified as *Briareum violaceum,* was screened, resulting in the isolation of eight natural briaranes, including three new briarines, briavioids E–G (**1**–**3**), and five known analogues, briacavatolides B and C, briaexcavatin L, briaexcavatolide U and briarenol K. The structures of all isolated compounds were determined using spectroscopic methods. An in vitro pro-inflammatory assay was also performed to evaluate the ability of briaranes **1** and **2** and **4**–**8** against the expression of iNOS and COX-2 proteins in LPS-induced RAW 264.7 macrophage cells. The anti-inflammatory activity results are shown in Table 2, and the structure–activity relationships (SAR) among some similar briaranes were also discussed. In addition, the absolute configuration of briavioid A (**9**) was reported in this study by using a single-crystal X-ray diffraction analysis with a copper radiation source, using the material obtained in previous study [12].

## Figures and Tables

**Table 1:** ^1^H and ^13^C NMR data for briavioids E–G (**1**–**3**) in CDCl_3_ at 25 °C.

	1		2		3	
C/H	*δ*_H_, ^a^ (*J* in Hz)	*δ*_C_, ^a^ mult. ^c^	*δ*_H_, ^a^ (*J* in Hz)	*δ*_C_, ^a^ mult. ^c^	*δ*_H_, ^b^ (*J* in Hz)	*δ*_C_, ^b^ mult. ^c^
1		47.8, C		44.2, C		44.2, C
2	4.97 d (8.4)	73.1, CH	3.88 s	85.2, CH	3.89 s	85.2, CH ^d^
3α	2.04 ddd (15.2, 8.4, 5.6)	37.6, CH_2_	5.71 d (7.2)	71.6, CH	5.72 d (7.2)	71.5, CH ^d^
β	2.97 dd (15.2, 12.4)					
4	4.99 dd (12.4, 5.6)	68.9, CH	6.32 d (7.2)	66.0, CH	6.31 d (7.2)	66.2, CH
5		140.9, C		137.7, C		137.5, C
6	5.48 ddd (8.4, 1.6, 1.6)	122.9, CH	5.65 dd (10.0, 1.2)	126.1, CH	5.66 dd (10.2, 1.8)	126.2, CH ^d^
7	5.60 d (8.4)	73.4, CH	5.71 d (10.0)	71.6, CH	5.71 d (10.2)	71.6, CH
8		70.4, C		71.1, C		71.0, C ^d^
9	5.80 d (1.6)	67.1, CH	3.94 dd (6.4, 5.6)	69.3, CH	3.95 dd (9.6, 6.0)	69.3, CH
10	2.12 d (1.6)	48.9, CH	3.15 br s	42.7, CH	3.15 br s	42.7, CH
11		78.2, C		134.3, C		134.2, C
12	3.75 ddd (12.4, 4.4, 2.8)	73.1, CH	5.34 dd (5.2, 0.8)	119.6, CH	5.35 dd (4.8, 1.2)	119.6, CH
13α	2.02 ddd (14.4, 4.4, 3.6)	30.2, CH_2_	2.05 m	28.4, CH_2_	2.04 m	28.4, CH_2_
β	1.67 ddd (14.4, 12.4, 2.0)		2.45 m		2.45 m	
14	4.83 dd (3.6, 2.0)	74.6, CH	4.60 t (2.4)	77.2, CH	4.60 br s	77.1, CH ^d^
15	1.24 s	14.4, CH_3_	1.33 s	20.3, CH_3_	1.34 s	20.2, CH_3_
16a	4.76 dd (15.6, 1.6)	65.7, CH_2_	2.02 d (1.2)	18.2, CH_3_	2.03 d (1.8)	18.2, CH_3_
b	5.25 dd (15.6, 1.6)					
17		66.1, C		61.3, C		61.2, C
18	1.79 s	10.2, CH_3_	1.62 s	9.3, CH_3_	1.62 s	9.3, CH_3_
19		170.1, C		172.1, C		172.1, C ^d^
20	1.17 s	17.0, CH_3_	1.74 d (0.8)	24.9, CH_3_	1.74 d (1.2)	24.9, CH_3_
OAc-2		170.5, C				
	2.01 s	21.3, CH_3_				
OAc-3				168.9, C		168.9, C ^d^
			2.03 s	20.7, CH_3_	2.05 s	20.6, CH_3_
OAc-4		169.8, C				171.7, C ^d^
	2.05 s	20.8, CH_3_			2.12 s	20.6, CH_3_
OAc-9		167.9, C				
	2.25 s	21.3, CH_3_				
OAc-14		170.4, C		170.7, C		170.7, C ^d^
	2.03 s	21.1, CH_3_	2.09 s	22.0, CH_3_	2.10 s	22.0, CH_3_
OAc-16		170.0, C				
	2.10 s	21.0, CH_3_				
OC(O)(CH_2_)_2_CH_3_-4				174.3, C		
			2.37 dt (16.4, 7.6)	35.8, CH_2_		
			2.28 dt (16.4, 7.6)			
			1.65 sext (7.6)	18.1, CH_2_		
			0.97 t (7.6)	13.7, CH_3_		
OH-2			5.38 br s		5.23 br s	
OH-9			6.45 br d (6.4)		6.42 d (9.6)	
OH-11	1.58 s					
OH-12	2.14 d (2.8)					

^a^ Data recorded at 400 MHz for *δ*_H_ and 100 MHz for *δ*_C_. ^b^ Data recorded at 600 MHz for *δ*_H_ and 150 MHz for *δ*_C_. ^c^ Multiplicity deduced from DEPT and HSQC spectra. ^d^ Data assigned with the assistance of HSQC and HMBC spectra.

**Table 2:** Effects of briaranes **1** and **2** and **4**–**8** (10 μM) on the expression of LPS-induced, pro-inflammatory iNOS and COX-2 proteins in macrophages.

	iNOS	COX-2		β-Actin
Compound	Expression (% of LPS)			
Control	0.04 ± 0.01	0.79	± 0.49	95.66 ± 3.25
LPS	100.00 ± 0.00	100.00	± 0.00	100.00 ± 0.00
Briavioid E (1)	77.16 ± 6.62	110.57	± 10.75	100.84 ± 1.61
Briavioid F (2)	51.60 ± 4.93	140.73	± 9.33	97.85 ± 5.27
Briacavatolide B (4)	62.93 ± 4.24	102.54	± 5.40	91.28 ± 5.30
Briacavatolide C (5)	69.12 ± 2.88	113.85	± 9.19	93.01 ± 7.84
Briaexcavatin L (6)	94.67 ± 6.77	114.65	± 11.73	103.35 ± 4.48
Briaexcavatolide U (7)	75.89 ± 3.70	121.70	± 8.93	99.81 ± 4.66
Briarenol K (8)	66.53 ± 3.92	123.02	± 10.46	91.45 ± 9.56
Dexamethasone	47.27 ± 2.22	30.36	± 1.73	97.71 ± 7.18

Data were normalized to the cells treated with LPS alone, and cells treated with dexamethasone (10 μM) were used as a positive control. Data are expressed as the mean ± SEM (*n* = 3).

## Data Availability

The data presented in this study are available in this article and the Supplementary Materials.

## Supplementary Materials

The following supporting information can be downloaded at: https://www.mdpi.com/article/10.3390/md21020124/s1, Figures S1–S29: ESIMS, HRESIMS, IR, 1D (^1^H, ^13^C and DEPT) and 2D (HSQC, HMBC, COSY and NOESY) NMR spectra of **1**–**3**.
