# Supplementary material for: Briavioids E–G, Newly Isolated Briarane-Diterpenoids from a Cultured Octocoral Briareum violaceum
Source: Mar Drugs. 2023 Feb 14;21(2):124. doi: 10.3390/md21020124 (PMC9959093; doi:10.3390/md21020124)
Supplement: Supplementary file 1 [file marinedrugs-21-00124-s001.zip › marinedrugs-2205693-supplementary.pdf]

Supporting information

S1. ESIMS spectrum of compound **1** 2
S2. HRESIMS spectrum of compound **1** 2
S3. IR spectrum of compound **1** 3
S4. ^{1}H NMR spectrum (400 MHz) of compound **1** in $CDCl_3$ 3
S5. ^{13}C NMR spectrum (100 MHz) of compound **1** in $CDCl_3$ 4
S6. DEPT spectra of compound **1** in $CDCl_3$ 4
S7. HSQC spectrum of compound **1** in $CDCl_3$ 5
S8. HMBC spectrum of compound **1** in $CDCl_3$ 5
S9. ^{1}H-^{1}H COSY spectrum of compound **1** in $CDCl_3$ 6
S10. NOESY spectrum of compound **1** in $CDCl_3$ 6
S11. ESIMS spectrum of compound **2** 7
S12. HRESIMS spectrum of compound **2** 7
S13. IR spectrum of compound **2** 8
S14. ^{1}H NMR spectrum (400 MHz) of compound **2** in $CDCl_3$ 8
S15. ^{13}C NMR spectrum (100 MHz) of compound **2** in $CDCl_3$ 9
S16. DEPT spectra of compound **2** in $CDCl_3$ 9
S17. HSQC spectrum of compound **2** in $CDCl_3$ 10
S18. HMBC spectrum of compound **2** in $CDCl_3$ 10
S19. ^{1}H-^{1}H COSY spectrum of compound **2** in $CDCl_3$ 11
S20. NOESY spectrum of compound **2** in $CDCl_3$ 11
S21. ESIMS spectrum of compound **3** 12
S22. HRESIMS spectrum of compound **3** 12
S23. IR spectrum of compound **3** 13
S24. ^{1}H NMR spectrum (600 MHz) of compound **3** in $CDCl_3$ 13
S25. ^{13}C NMR spectrum (150 MHz) of compound **3** in $CDCl_3$ 14
S26. HSQC spectrum of compound **3** in $CDCl_3$ 14
S27. HMBC spectrum of compound **3** in $CDCl_3$ 15
S28. ^{1}H-^{1}H COSY spectrum of compound **3** in $CDCl_3$ 15
S29. NOESY spectrum of compound **3** in $CDCl_3$ 16

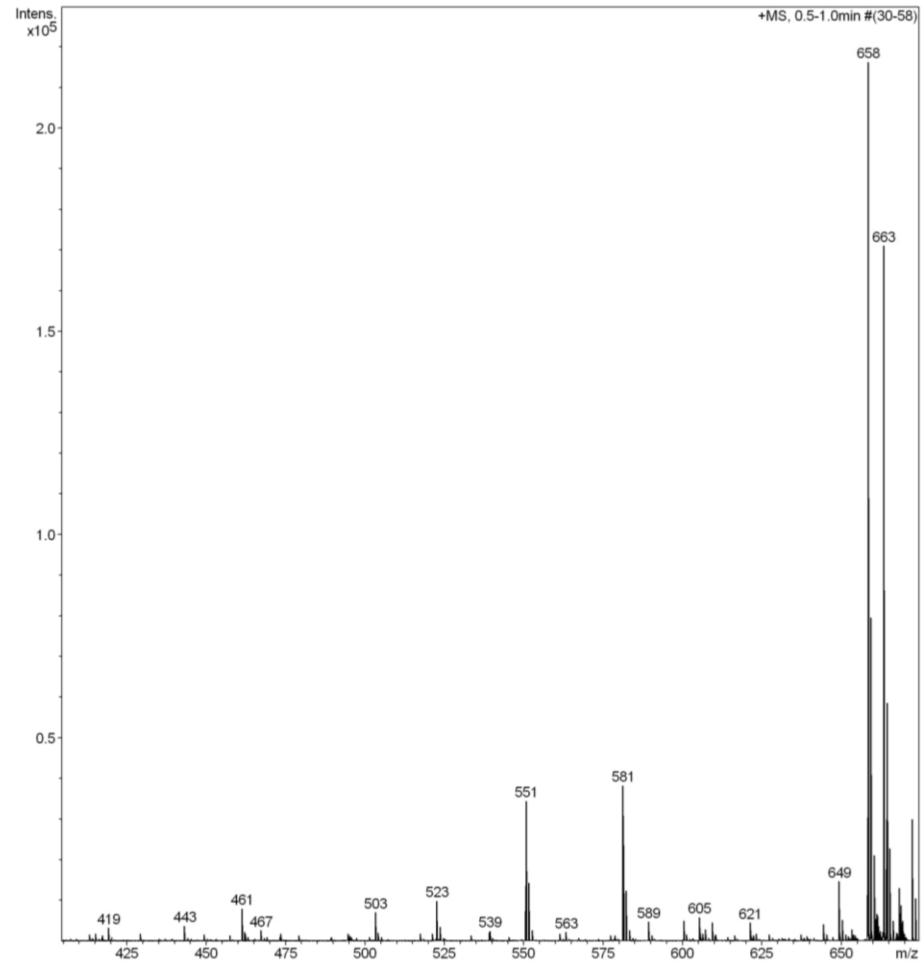

S1. ESIMS spectrum of compound **1**

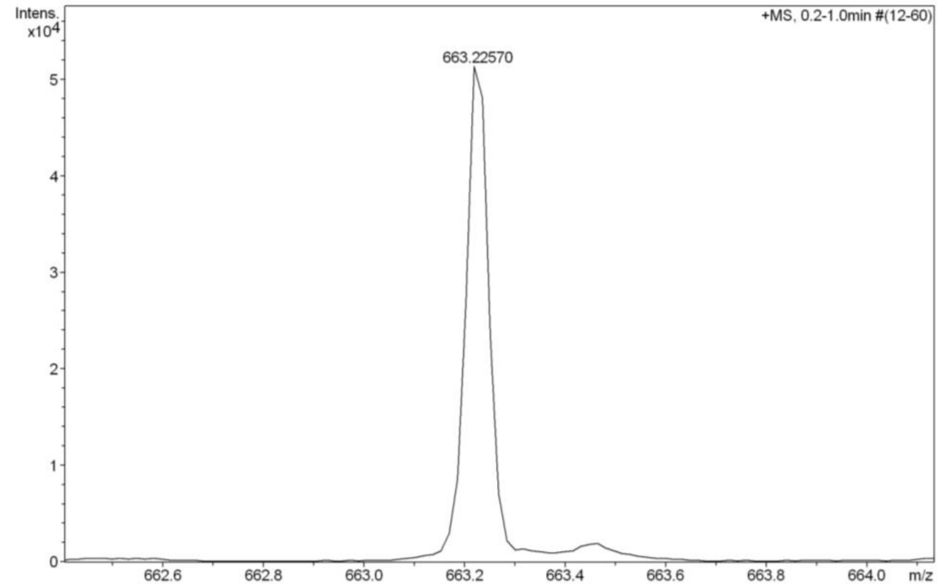

Meas. m/z	#	Formula	Score	m/z	err [mDa]	err [ppm]	mSigma	rdb	e⁻ Conf	N-Rule
663.22570	1	C 30 H 40 Na O 15	100.00	663.22594	0.24	0.36	39.2	10.5	even	ok

S2. HRESIMS spectrum of compound **1**

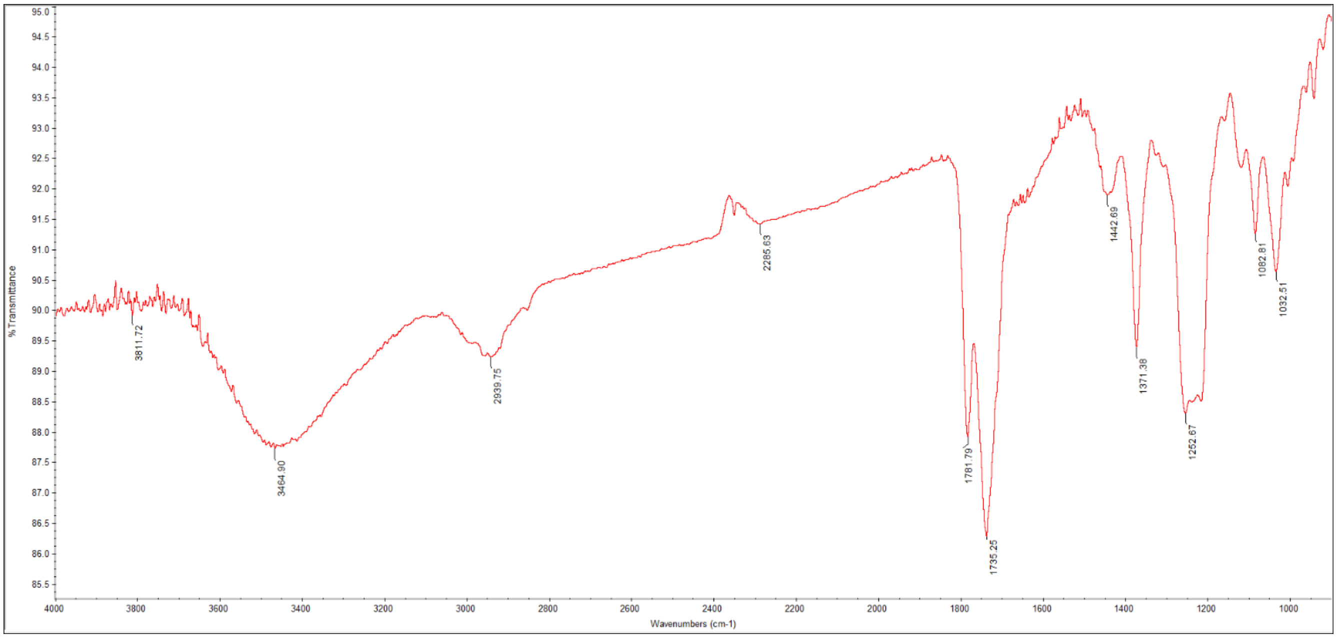

S3. IR spectrum of compound **1**

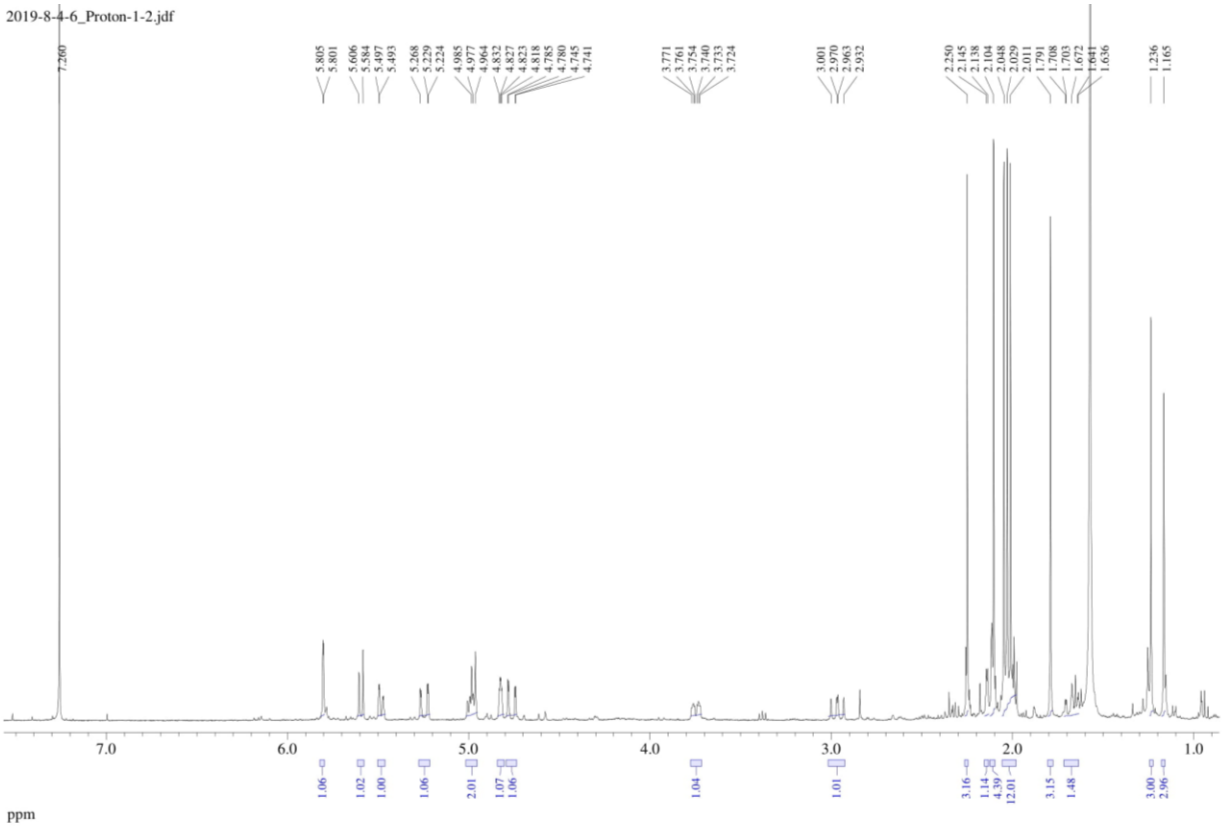

S4. ^{1}H NMR spectrum (400 MHz) of compound **1** in $CDCl_3$

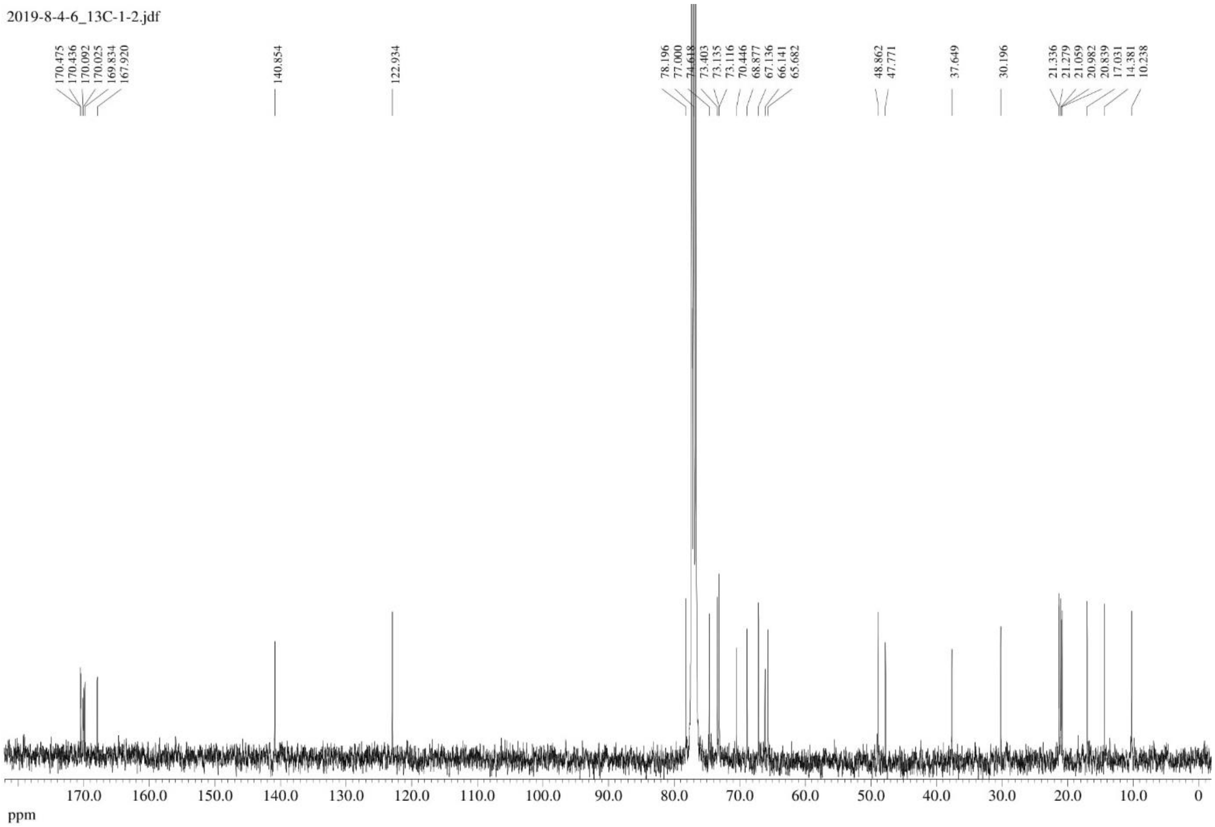

S5. ^{13}C NMR spectrum (100 MHz) of compound **1** in $CDCl_3$

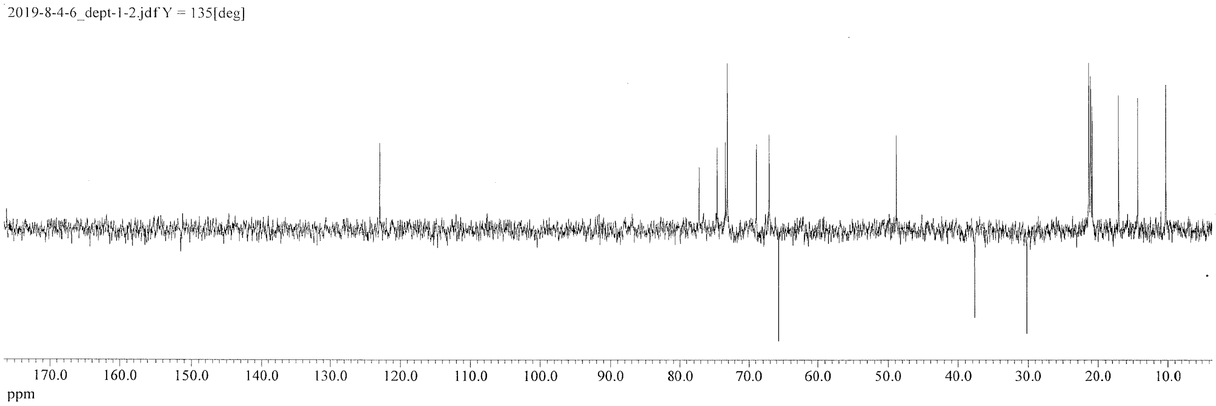

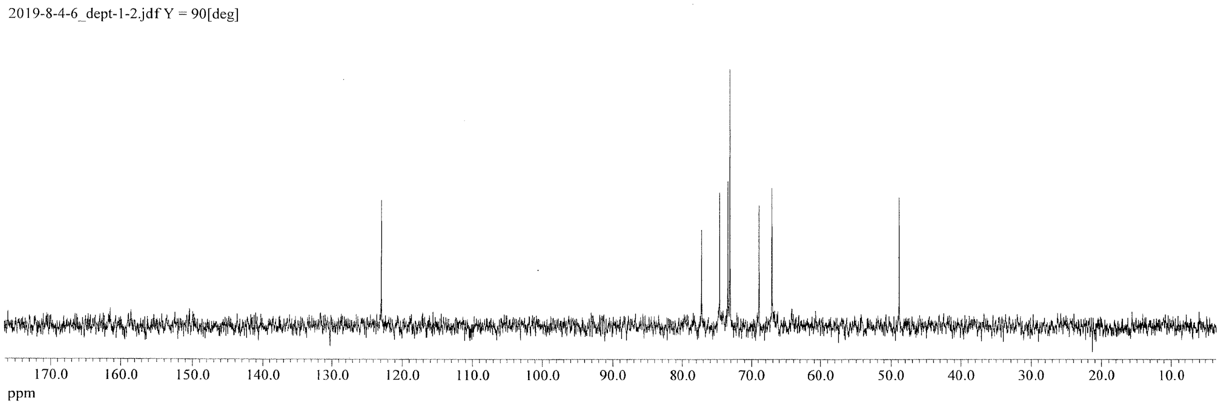

S6. DEPT spectra of compound **1** in $CDCl_3$

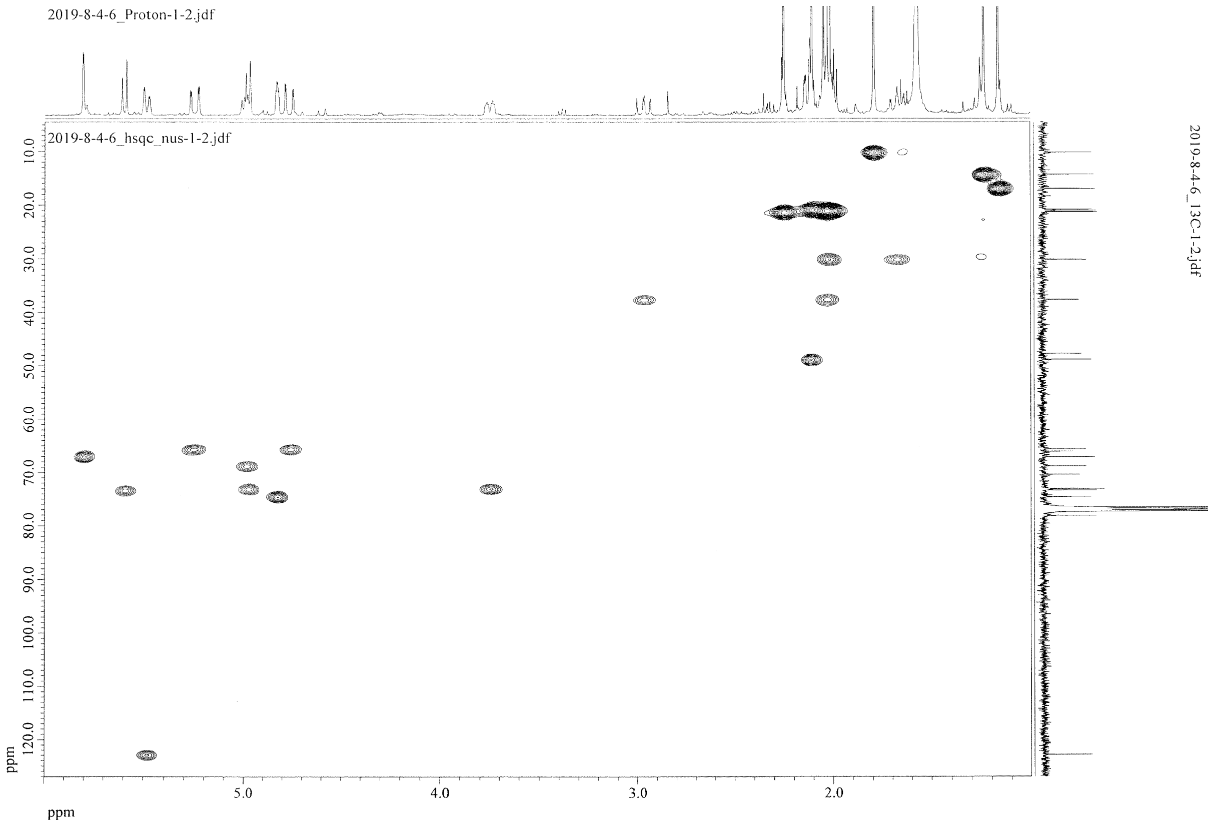

S7. HSQC spectrum of compound **1** in $CDCl_3$

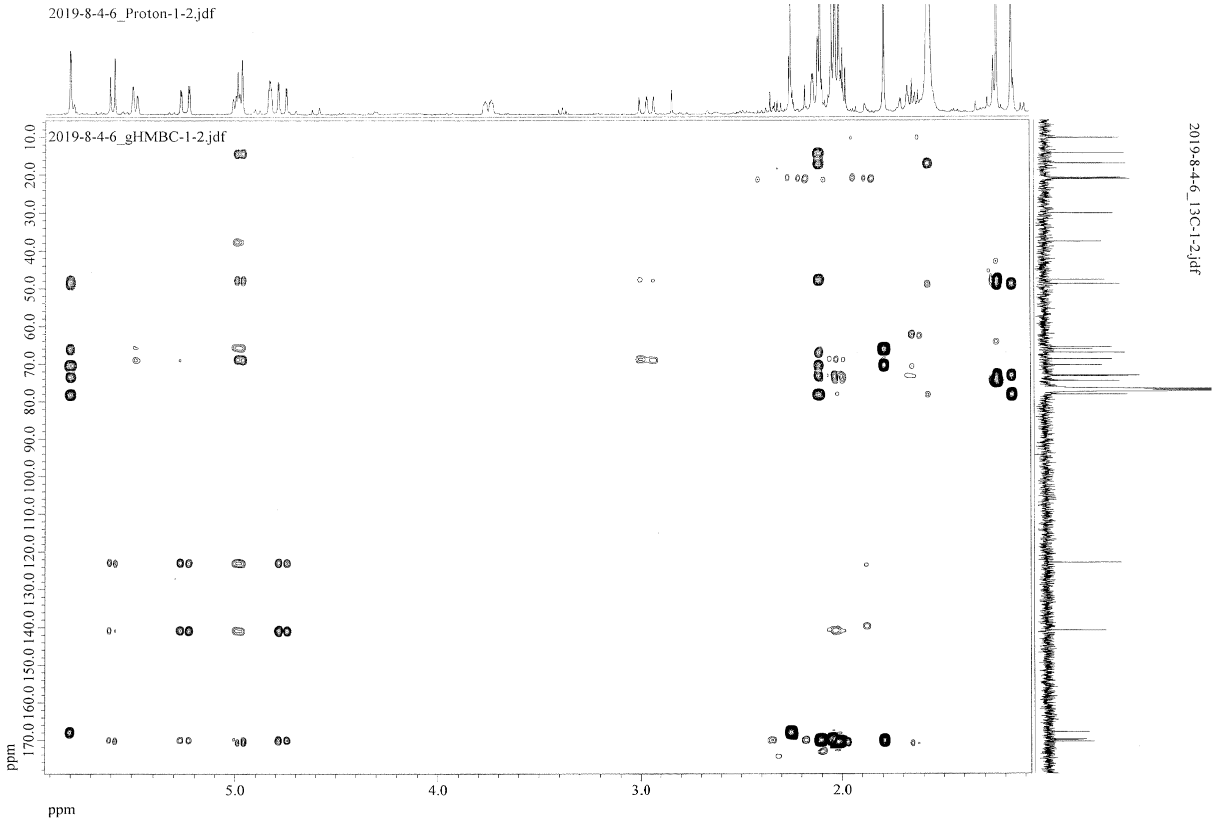

S8. HMBC spectrum of compound **1** in $CDCl_3$

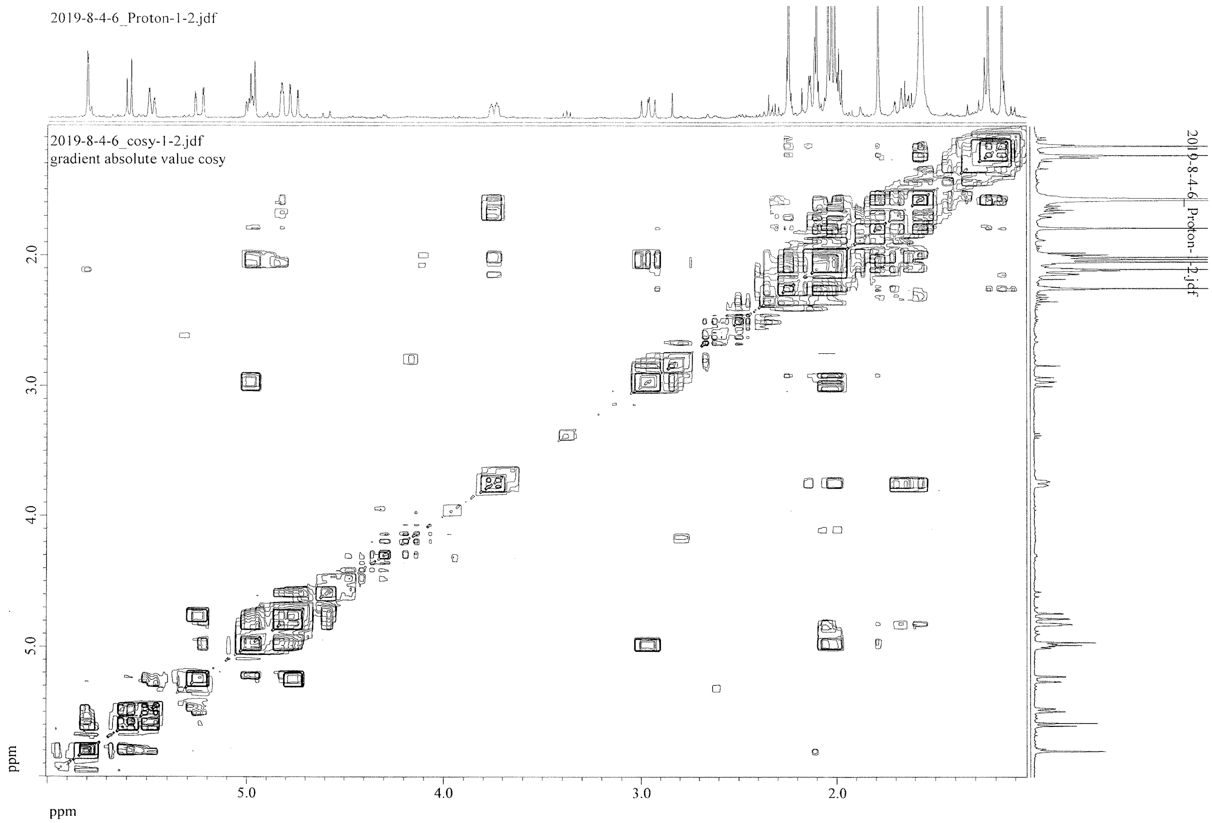

S9. ^{1}H-^{1}H COSY spectrum of compound **1** in $CDCl_3$

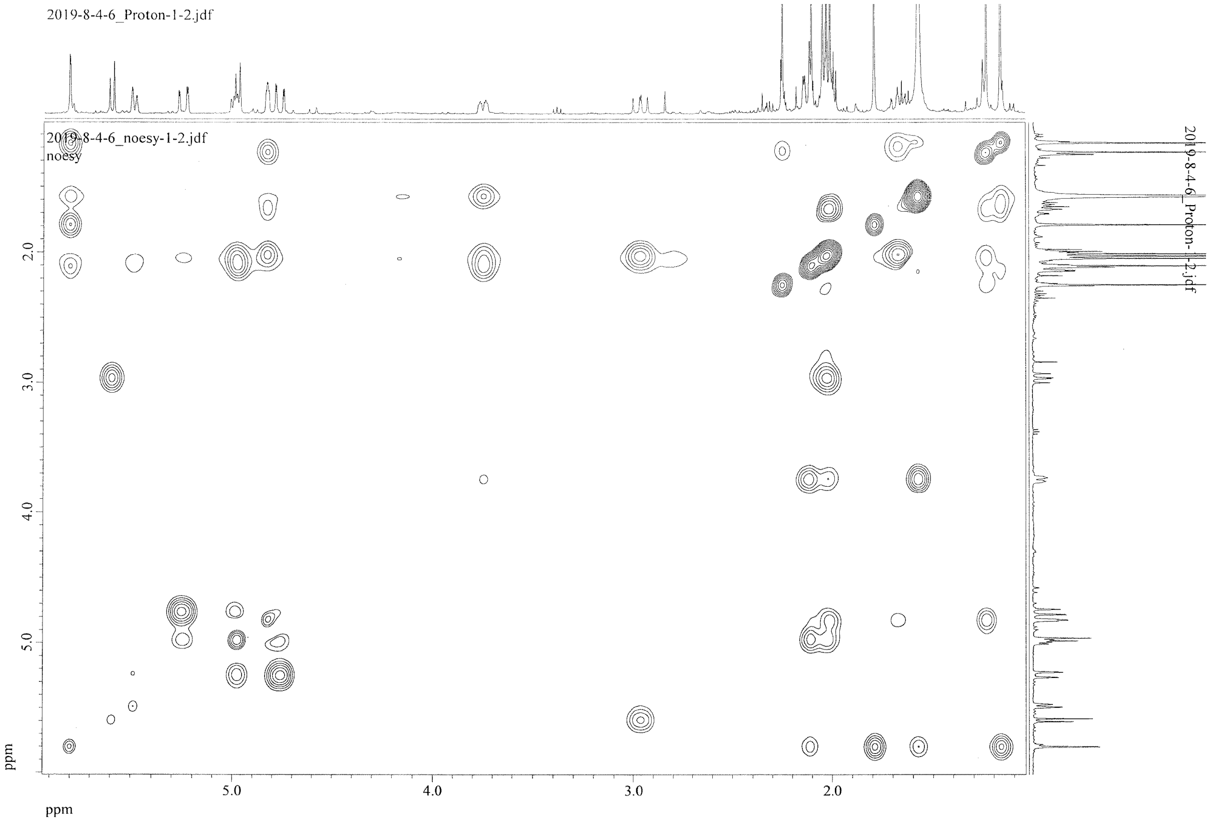

S10. NOESY spectrum of compound **1** in $CDCl_3$

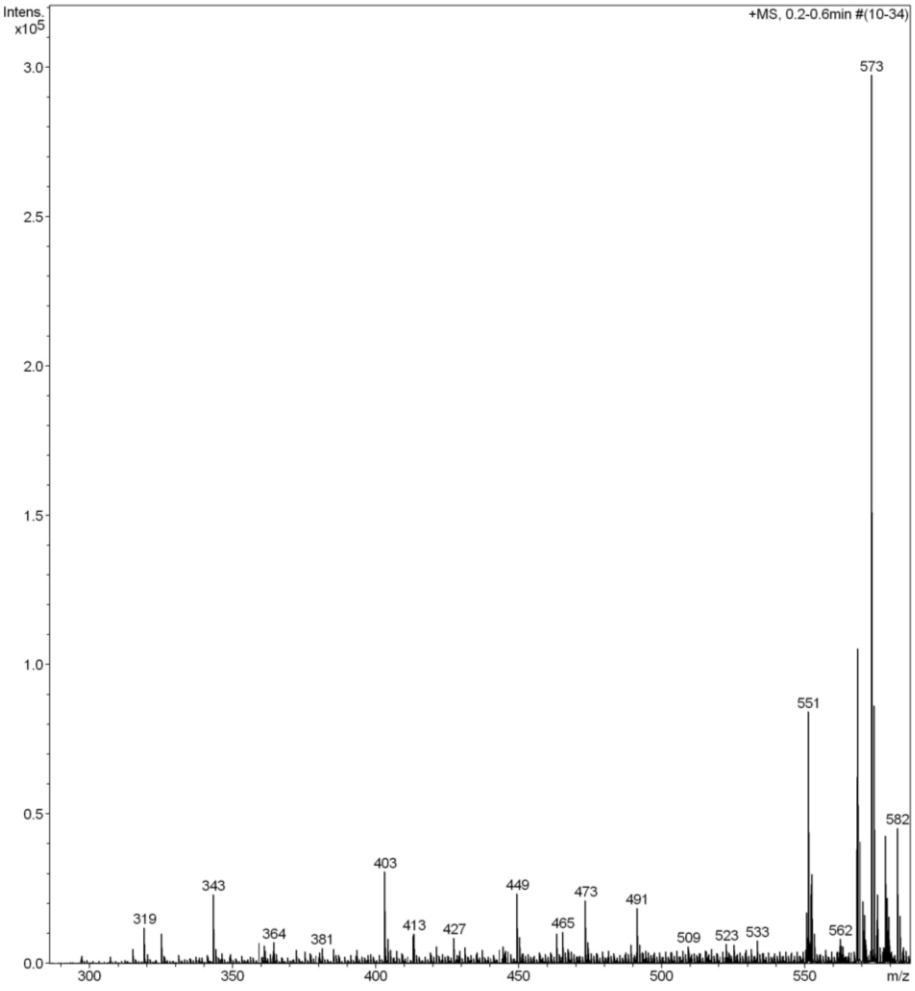

S11. ESIMS spectrum of compound **2**

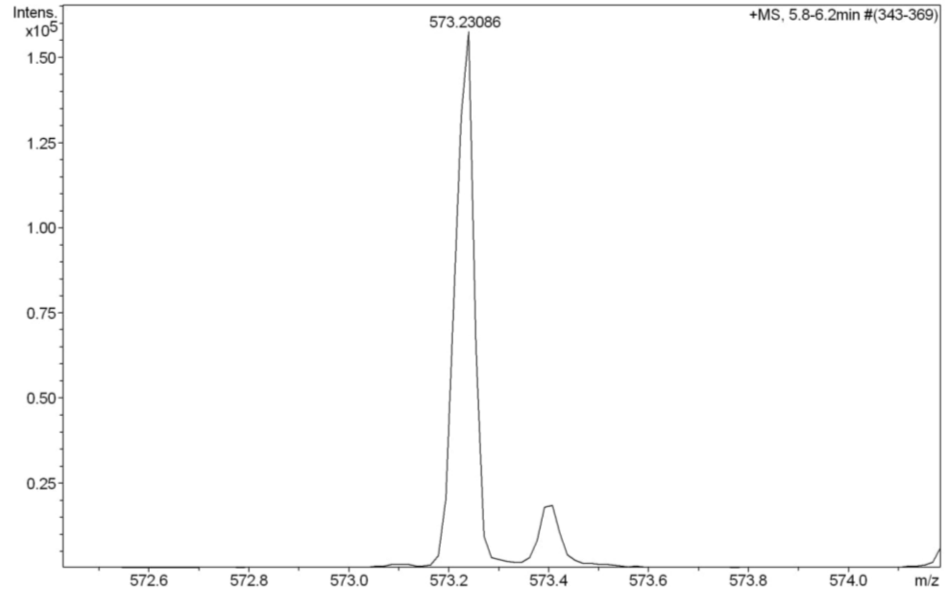

Meas. m/z	#	Formula	Score	m/z	err [mDa]	err [ppm]	mSigma	rdb	e⁻ Conf	N-Rule
573.23086	1	C 28 H 38 Na O 11	100.00	573.23063	-0.22	-0.39	21.1	9.5	even	ok

S12. HRESIMS spectrum of compound **2**

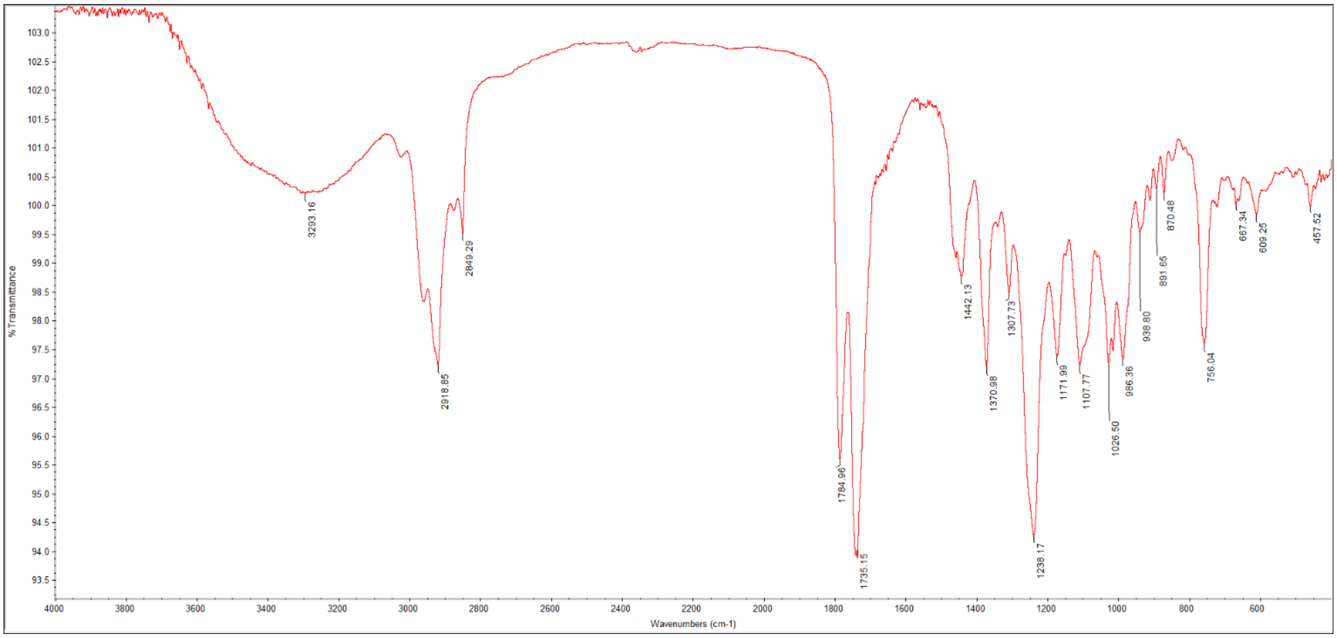

S13. IR spectrum of compound **2**

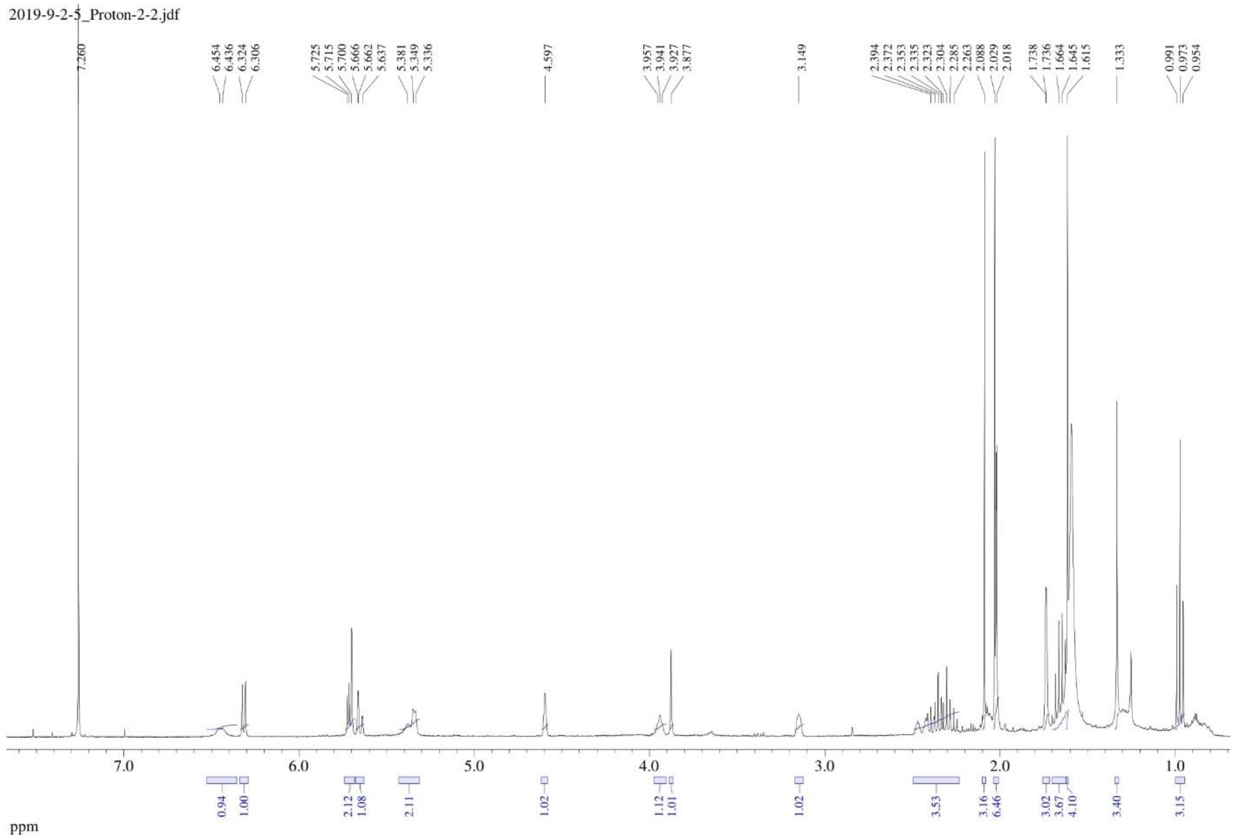

S14. ^{1}H NMR spectrum (400 MHz) of compound **2** in $CDCl_3$

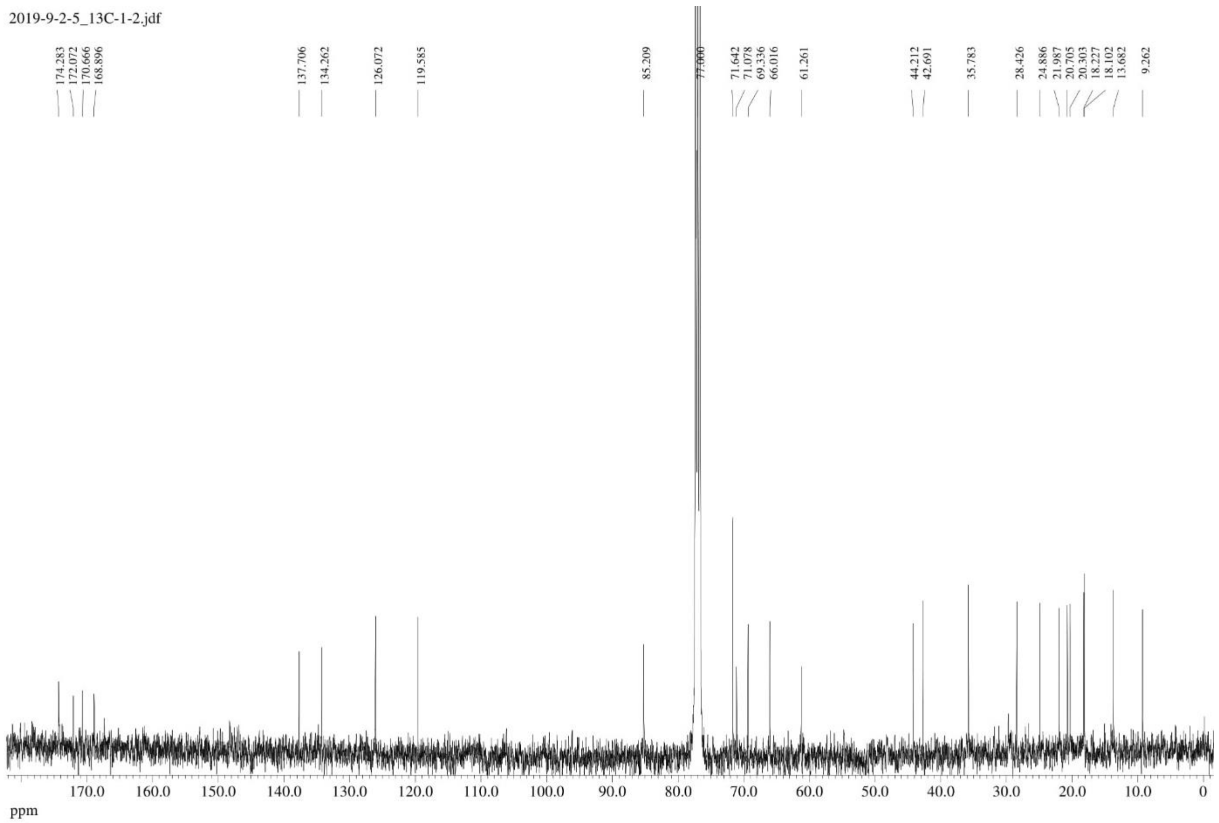

S15. ^{13}C NMR spectrum (100 MHz) of compound **2** in $CDCl_3$

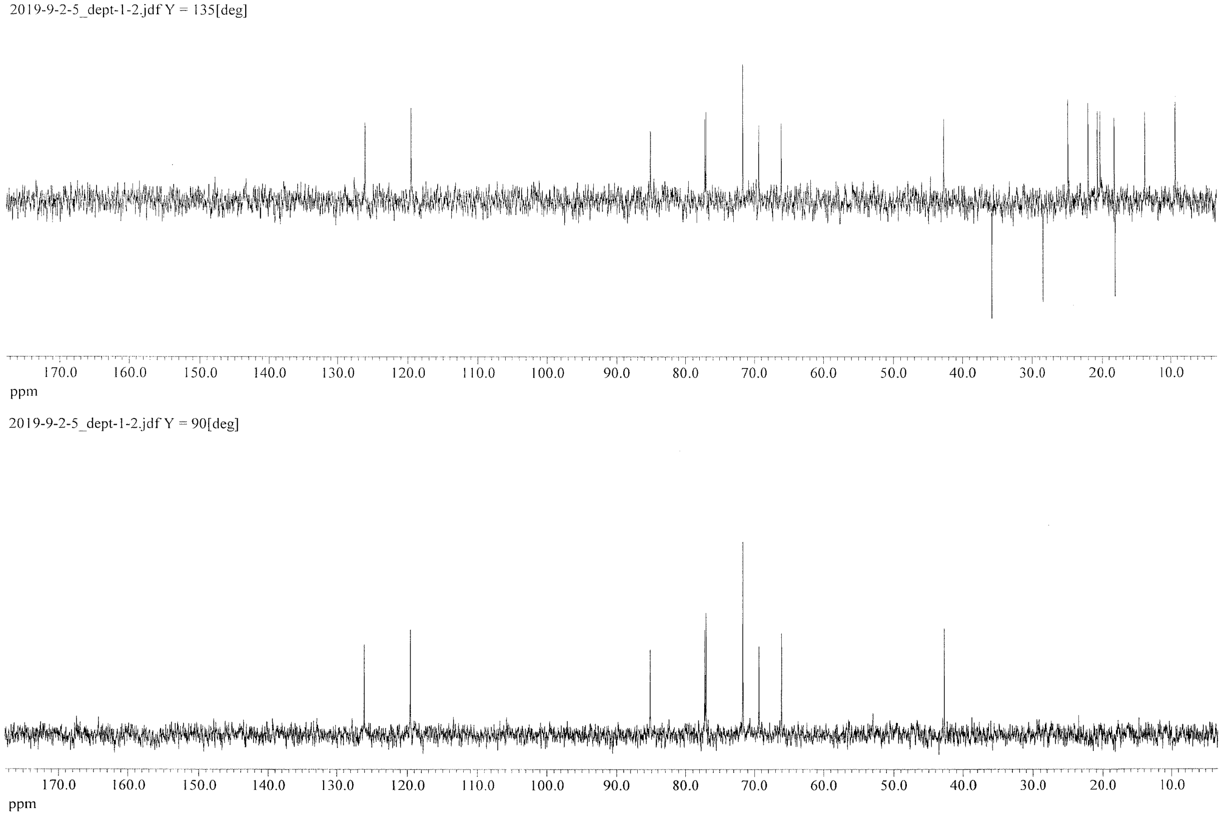

S16. DEPT spectra of compound **2** in $CDCl_3$

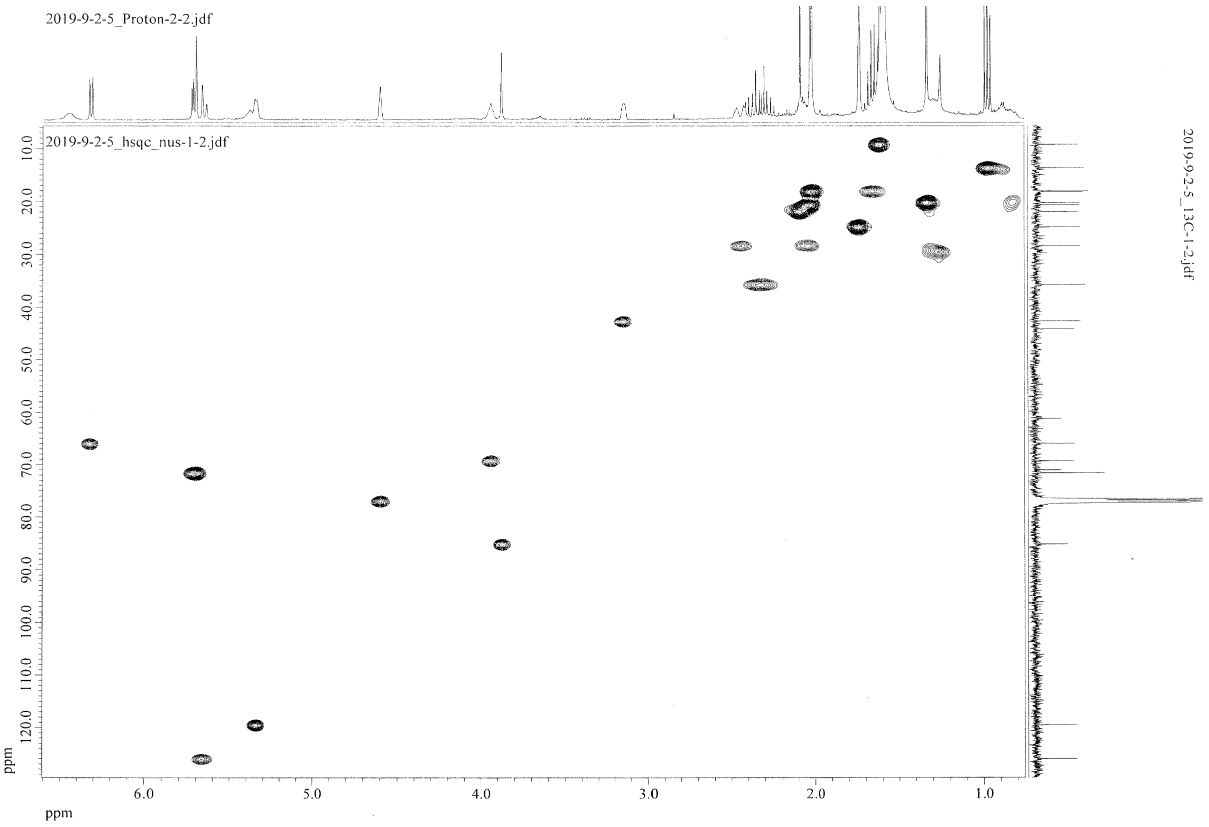

S17. HSQC spectrum of compound **2** in $CDCl_3$

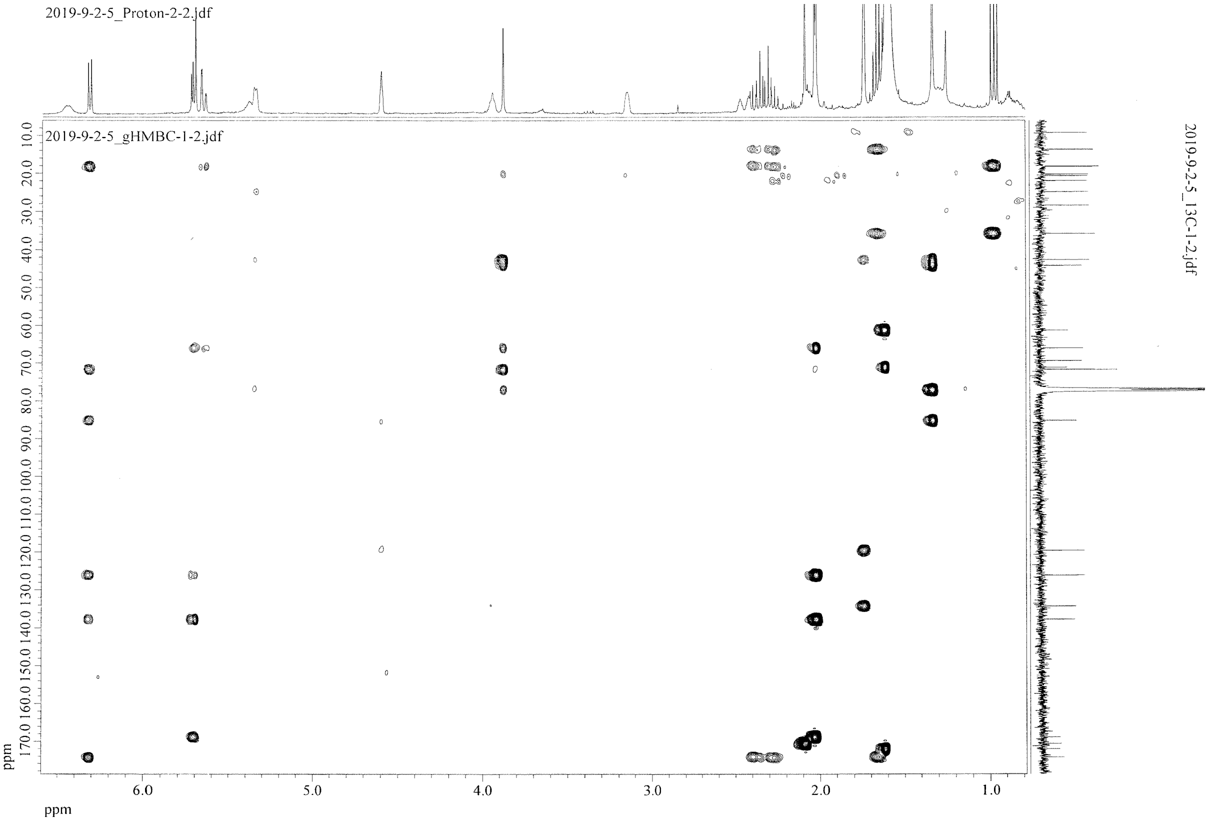

S18. HMBC spectrum of compound **2** in $CDCl_3$

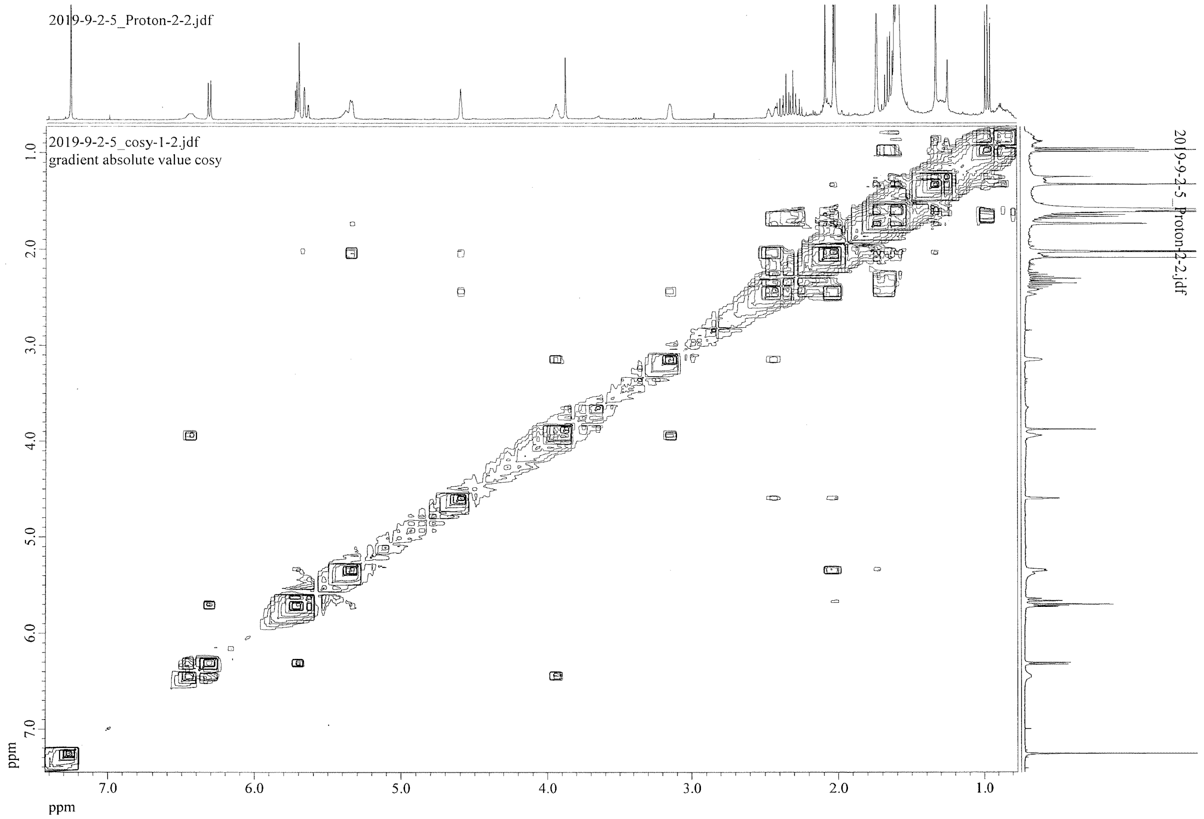

S19. ^{1}H-^{1}H COSY spectrum of compound **2** in $CDCl_3$

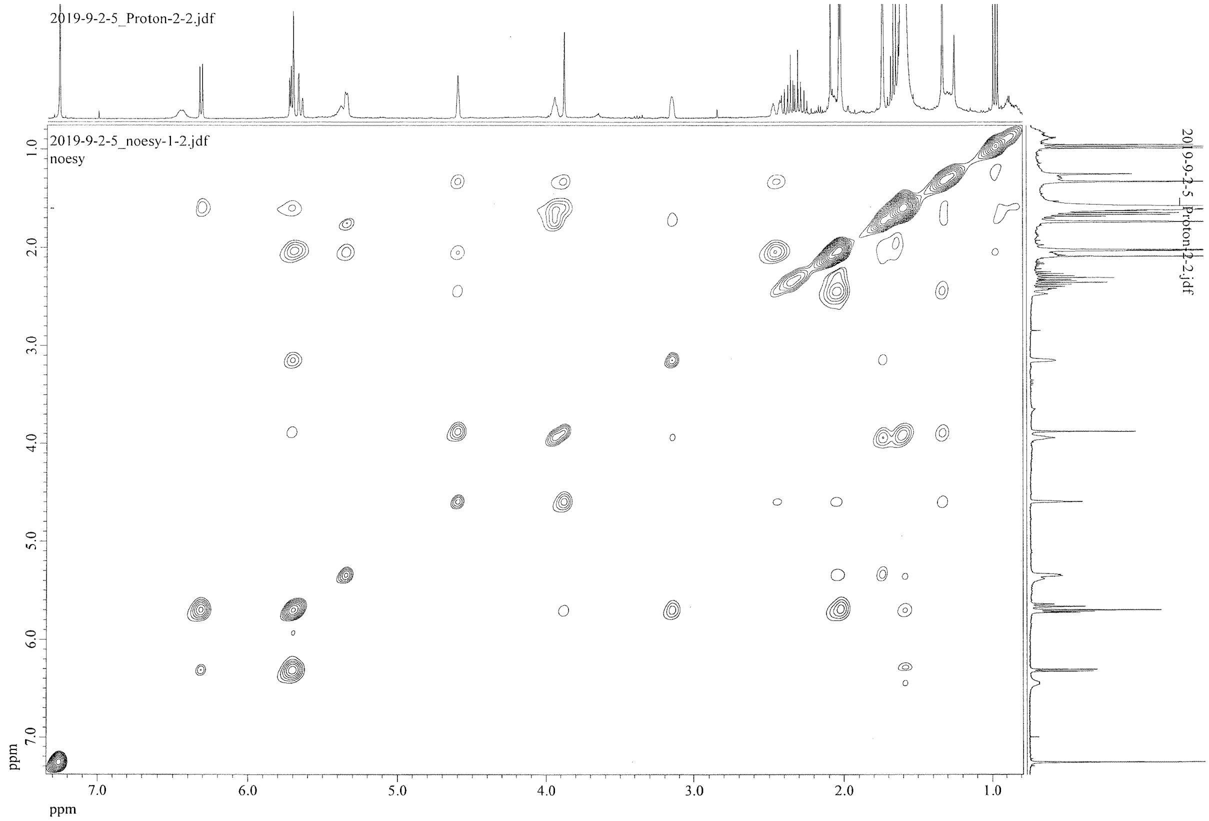

S20. NOESY spectrum of compound **2** in $CDCl_3$

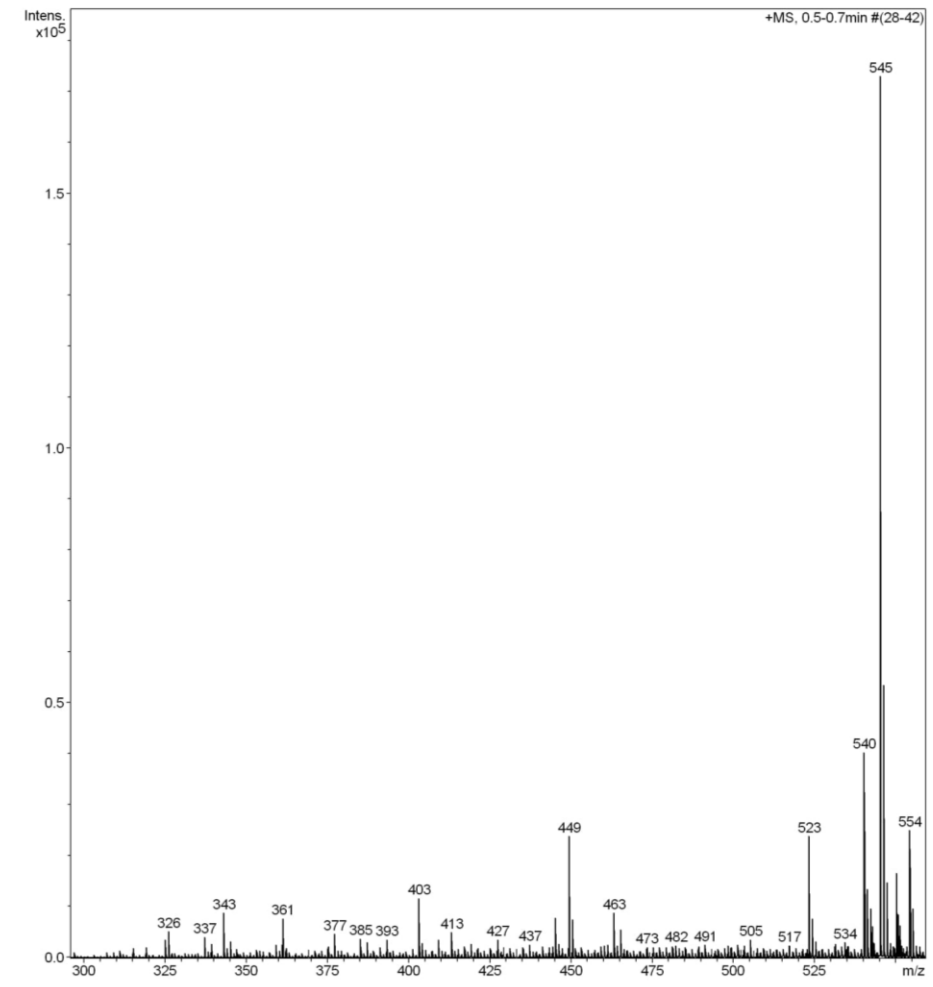

S21. HRESIMS spectrum of compound **3**

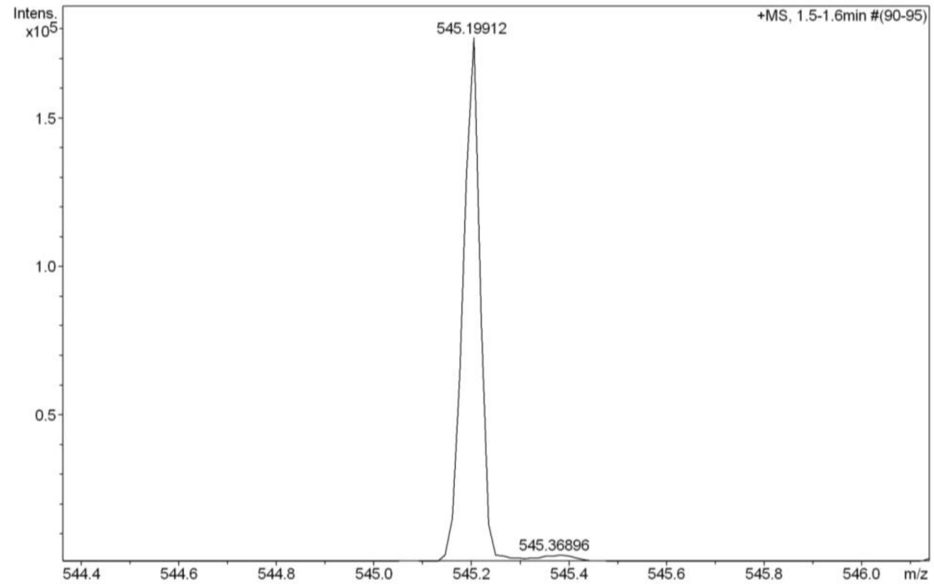

Meas. m/z	#	Formula	Score	m/z	err [mDa]	err [ppm]	mSigma	rdb	e⁻ Conf	N-Rule
545.19912	1	C 26 H 34 Na O 11	100.00	545.19933	0.21	0.39	16.6	9.5	even	ok

S22. HRESIMS spectrum of compound **3**

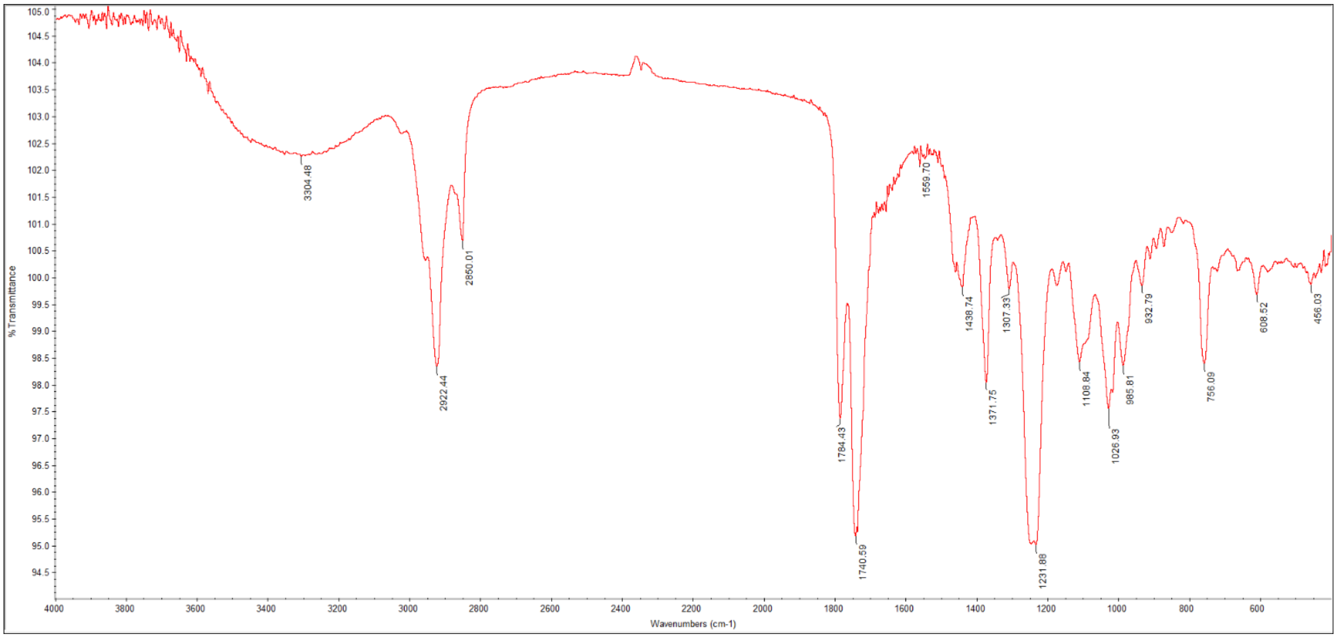

S23. IR spectrum of compound **3**

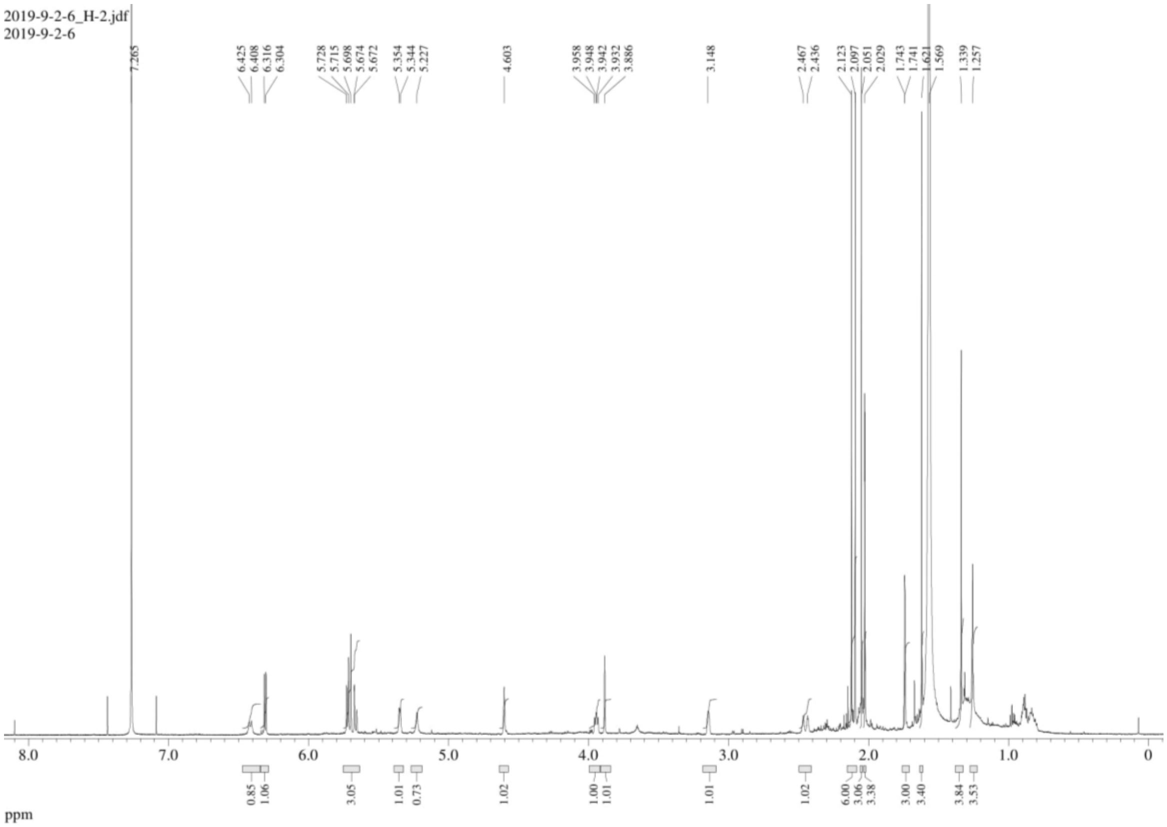

S24. ^{1}H NMR spectrum (600 MHz) of compound **3** in $CDCl_3$

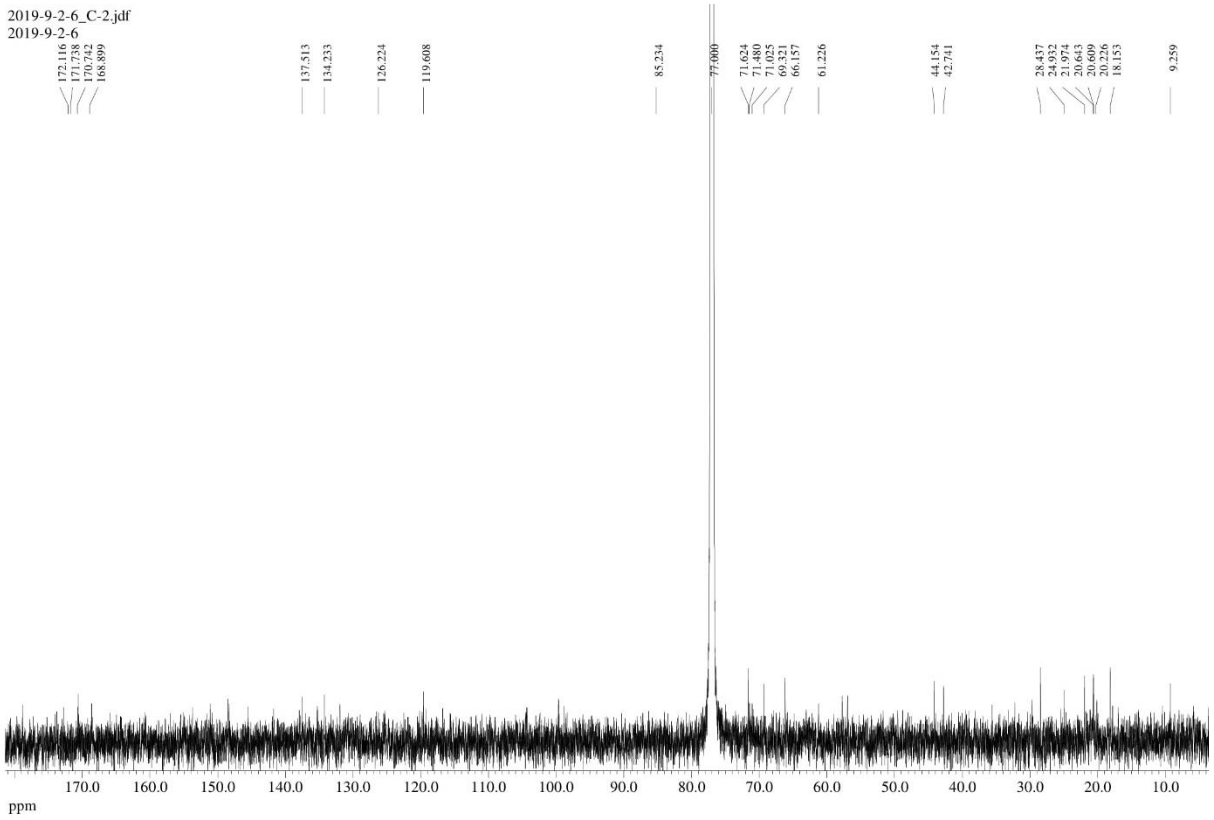

S25. ^{13}C NMR spectrum (150 MHz) of compound **3** in $CDCl_3$

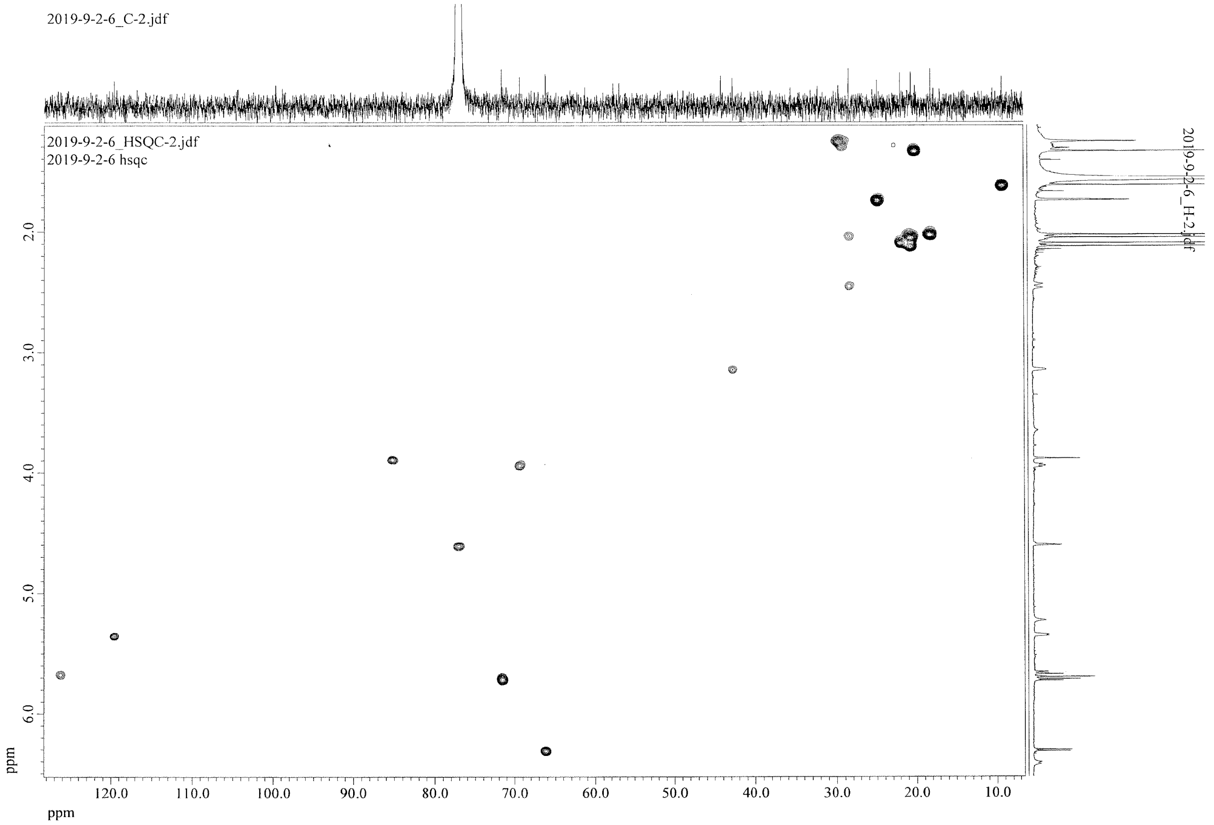

S26. HSQC spectrum of compound **3** in $CDCl_3$

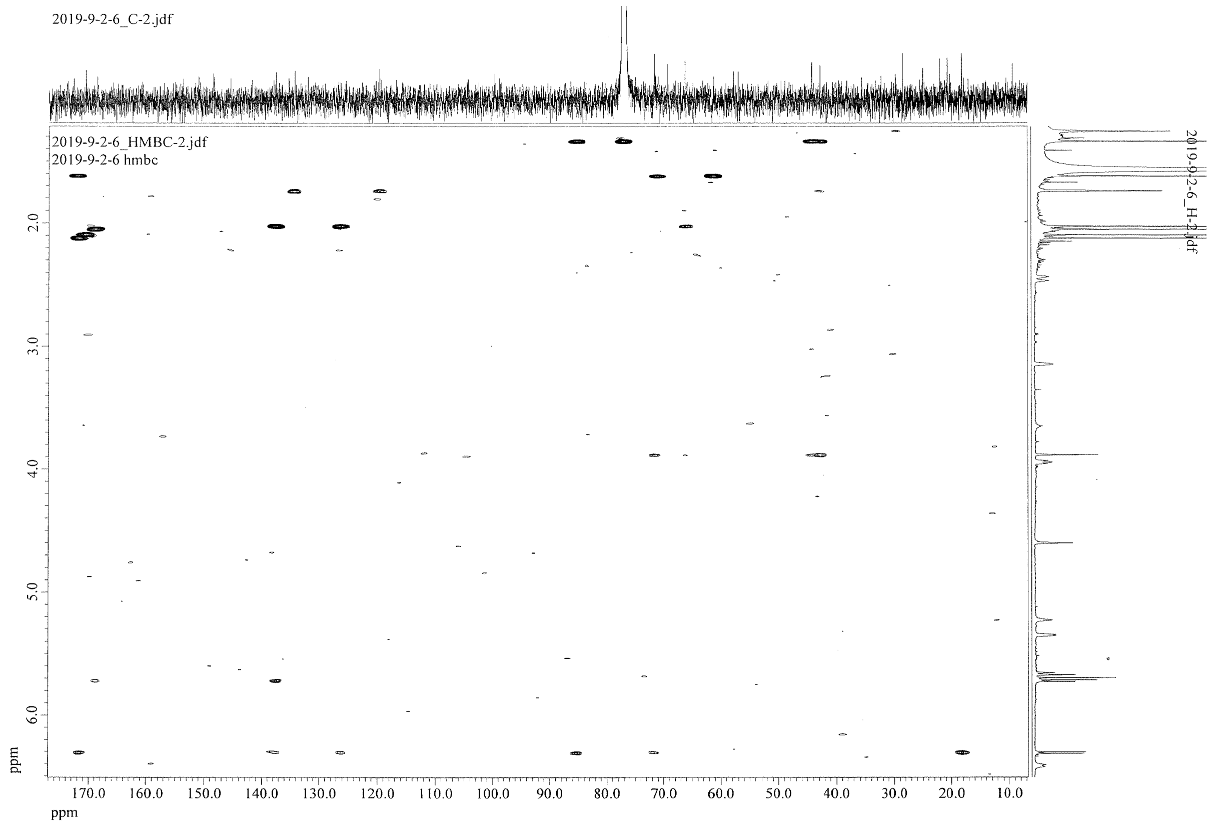

S27. HMBC spectrum of compound **3** in $CDCl_3$

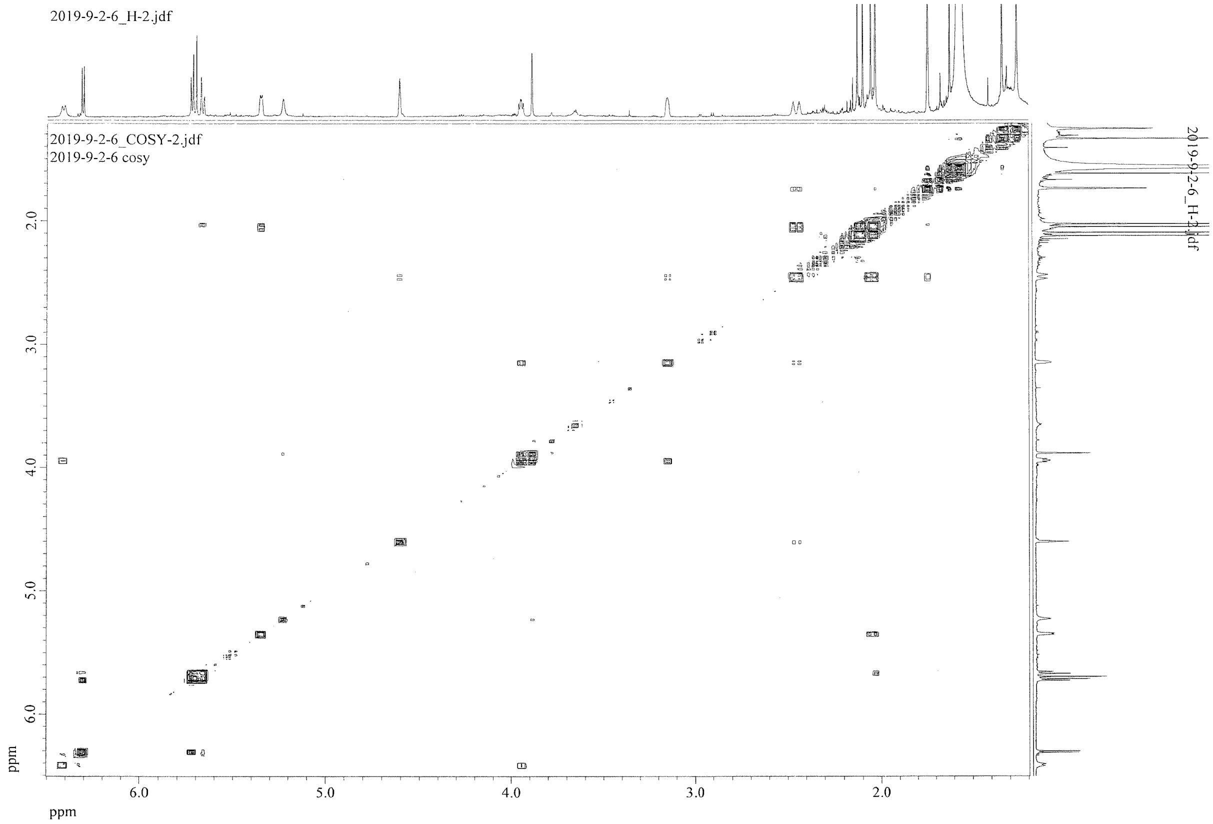

S28. ^1H-^1H COSY spectrum of compound **3** in $CDCl_3$

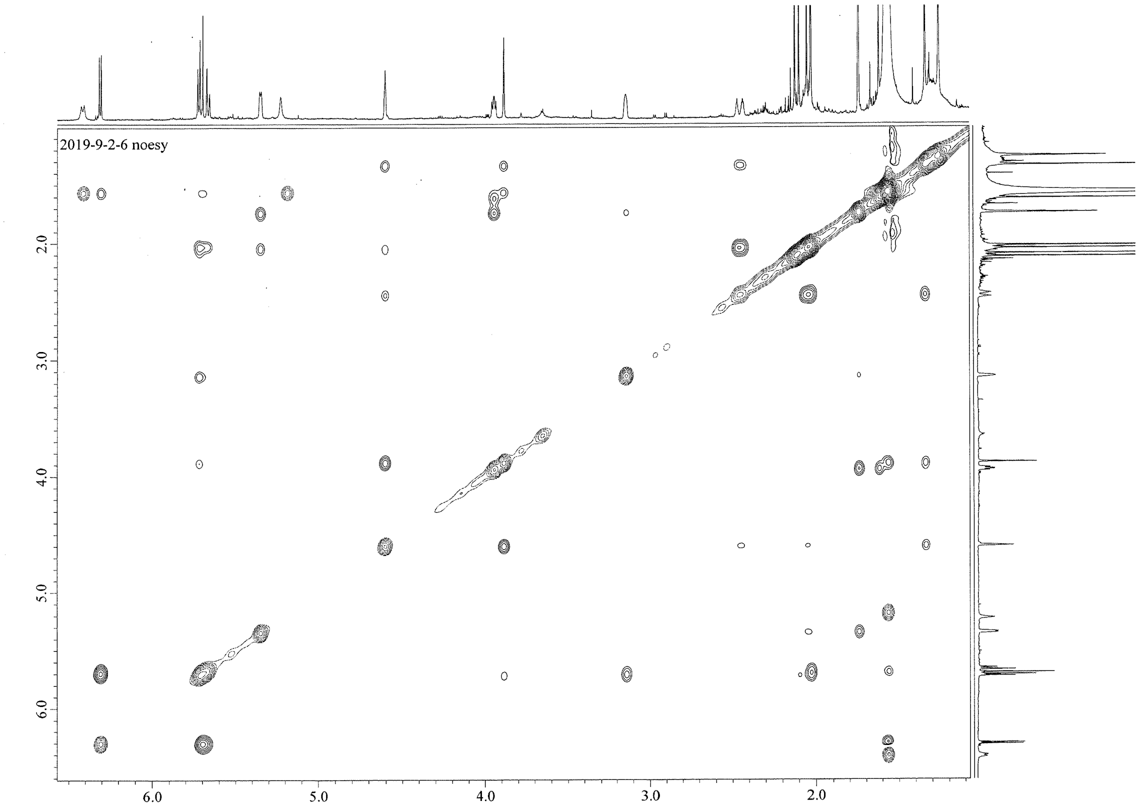

S29. NOESY spectrum of compound **3** in $CDCl_3$
